# Synchronous 500-year oscillations of monsoon climate and human activity in Northeast Asia

**DOI:** 10.1038/s41467-019-12138-0

**Published:** 2019-09-11

**Authors:** Deke Xu, Houyuan Lu, Guoqiang Chu, Li Liu, Caiming Shen, Fengjiang Li, Can Wang, Naiqin Wu

**Affiliations:** 1 Key Laboratory of Cenozoic Geology and Environment, Institute of Geology and Geophysics, Chinese Academy of Sciences, Beijing, 100029 China; 2 CAS Center for Excellence in Tibetan Plateau Earth Sciences, Beijing, 100101 China; 3 University of Chinese Academy of Sciences, Beijing, 100049 China; 4 CAS Center for Excellence in Life and Paleoenvironment, Beijing, 100044 China; 5 Department of East Asian Languages and Cultures, Stanford University, Stanford, CA 94305 USA; 6 Yunnan Key Laboratory of Plateau Geographical Processes and Environmental Changes, College of Tourism and Geographical Sciences, Yunnan Normal University, Kunming, 650500 China; 7 Institutions of Earth Science, Chinese Academy of Sciences, Beijing, 100029 China; 8 Department of Archaeology, School of History and Culture, Shandong University, Jinan, 250100 China

**Keywords:** Archaeology, Palaeoclimate, Palaeoecology, Palaeontology

## Abstract

Prehistoric human activities were likely influenced by cyclic monsoon climate changes in East Asia. Here we report a decadal-resolution Holocene pollen record from an annually-laminated Maar Lake in Northeast China, a proxy of monsoon climate, together with a compilation of 627 radiocarbon dates from archeological sites in Northeast China which is a proxy of human activity. The results reveal synchronous ~500-year quasi-periodic changes over the last 8000 years. The warm-humid/cold-dry phases of monsoon cycles correspond closely to the intensification/weakening of human activity and the flourishing/decline of prehistoric cultures. Six prosperous phases of prehistoric cultures, with one exception, correspond approximately to warm-humid phases caused by a strengthened monsoon. This ~500-year cyclicity in the monsoon and thus environmental change triggered the development of prehistoric cultures in Northeast China. The cyclicity is apparently linked to the El Niño-Southern Oscillation, against the background of long-term Holocene climatic evolution. These findings reveal a pronounced relationship between prehistoric human activity and cyclical climate change.

## Abstract (web summary)

Long-term climate cycles can potentially influence population dynamics, including those of humans. Here, the authors combine climate and archaeological records from Northeast China over the past 8000 years and demonstrate ~500 year cycles in both the monsoon and human activity.

## Introduction

**Fig. 1:**
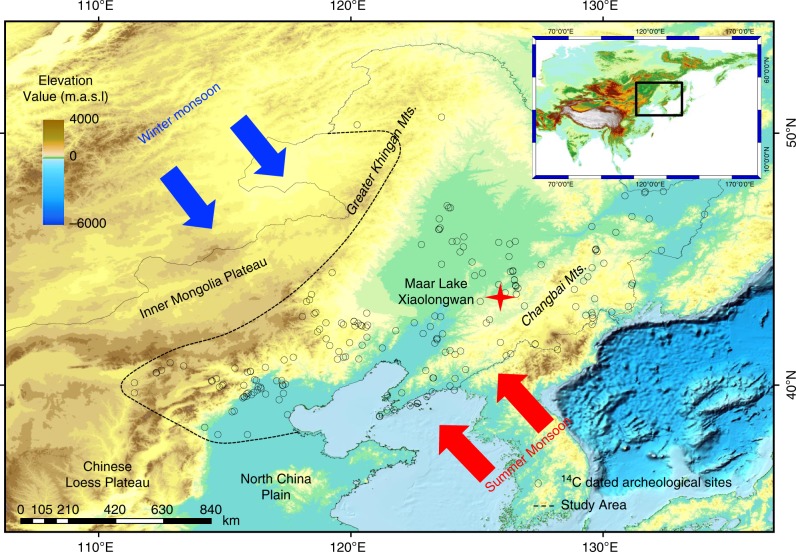
Locations of Maar Lake Xiaolongwan (red star) and radiocarbon dates from Neolithic archeological sites in NE China (open circles). Trajectories of the East Asian Summer Monsoon and Winter Monsoon are shown by red and blue arrows, respectively. This figure was generated using DIVA-GIS 7.5 (http://www.diva-gis.org/)

Monsoonal climate change at the decadal-centennial scale played a crucial role in influencing past and modern ecosystem evolution and human activities in East Asia^1,2^. The Holocene is one of the key time intervals for understanding the relationship between changes in the East Asian Summer Monsoon (EASM) and human activity. Holocene climate change can affect human society in two principal ways: (1) abrupt catastrophic climatic events, such as floods and droughts^3,4^, have major impacts, and it has been suggested that El Niño-Southern Oscillation (ENSO)^5^, and volcanic^6^ and solar activity^7^ may have played a key role in driving abrupt monsoon changes and human activity^7^; (2) cyclic climate changes, such as quasi-centennial oscillations, were likely associated with human developments such as nomadic migration and dynastic alternations in China^4,8^. However, studies^4,8^ of the latter have focused on the historical period, and the few that have focused on the early-middle Holocene are controversial^9^, mainly due to the lack of annual- to decadal-resolution climate records and quantitative proxies of human activity and cultural evolution. Here we report the results of a study of annually laminated Maar Lake Xiaolongwan (Fig. 1) (42°18.0′N, 126°21.5′E) in Northeast (NE) China, which has recently yielded a decadal-resolution pollen record spanning the past 5350 years (yr)^10,11^. This site provides an opportunity to establish a decadal-resolution climate record for the entire Holocene.

NE China is one of the regions where millet-based agriculture and early complex societies developed. The region has experienced six major Neolithic and Bronze Age cultures during the Holocene: Xinglongwa, Zhaobaogou, Hongshan, Xiaoheyan, Lower Xiajiadian, and Upper Xiajiadian. Archaeological investigations have demonstrated fluctuations in population density and settlement distribution through time^12,13^. The Neolithic Hongshan culture, especially, has attracted much attention in the study of Chinese civilization, because it developed prior to the emergence of dynastic civilization in the Central Plain. The Hongshan culture is characterized by large-scale public architecture and elite burials associated with the abundant use of jade at the ritual centers of Niuheliang and Dongshanzui sites. The disappearance of the Hongshan culture, often described as a rapid and total collapse due to climatic change, has also generated considerable speculation^14,15^. Despite the cultural importance of NE China, there has been no systematic evaluation using accurately dated quantitative records of human activity which are correlated with settlement patterns and cultural change in the region. In the last decade, an integrated dataset of archeological radiocarbon dates has been developed as a proxy for prehistoric human population, activity, and cultural change. It is based on the proposition that a larger population would result in the increased production and deposition of cultural carbon, thus providing more material evidence for age determinations^1,16–18^. This approach has been used to reconstruct early human activity and population history in different regions, including Europe, North America and China^1,16–18^, and therefore we used this proven approach to analyze the data from NE China.

Here, we present two sets of completely independent proxies: one is a decadal-resolution Holocene pollen record derived from an annually laminated Maar Lake Xiaolongwan, which is a proxy of monsoon climate; and the other is a dataset of integrated archaeological radiocarbon dates from NE China^1^, which is a proxy of human activity and prehistoric cultures. The results provide robust evidence for synchronous ~500-yr cyclical changes in monsoon climate, human activity and prehistoric cultural development in the East Asian Monsoon (EAM) region during the Holocene. Six prosperous phases of Neolithic and Bronze Age cultures correspond approximately to warm-humid phases caused by a strengthened EASM, except for the first expansion of the Hongshan culture, which corresponds to the phase of strongest EASM in the middle Holocene. We suggest that humans responded to climatic fluctuations with different social strategies, leading to the rise and fall of early complex societies in the region.

## Results

### Site description, sediment sampling and chronologies

Maar Lake Xiaolongwan is located in the Changbai Mountains, Jilin Province, NE China (Fig. 1 and Supplementary Fig. 1). The lake has a surface area of 0.079 km^2^, a maximum depth of 15 m, and a catchment area of 0.16 km^2^; it is a closed basin with no inflows or outflows^19^. The lake is dystrophic and humic, with brown-colored water which contains a high concentration of organic matter and has a low pH^20^. The lake has seasonal dinoflagellate blooms and is annually laminated^21^ (Supplementary Note 1). Broad-leaved deciduous forest (<720 m a.s.l), mixed deciduous and coniferous forest (720–1100 m a.s.l.) and coniferous forest (1100–1800 m a.s.l.) are present, with increasing altitude, in the mountainous area around the lake^11^. The climate of the region is controlled mainly by the EAM system^10,22^. High rainfall is usually coupled with high temperatures during the summer season^10,22^.

A 387-cm-long piston core was raised from a water depth of 14.5 m near the center of the lake in early spring 2006. Initial studies using scanning electron and optical microscopy, sediment trap observations, and an independent chronology (Methods) [lamination counting (Supplementary Fig. 2), and ^137^Cs, ^210^Pb (Supplementary Fig. 3) and AMS ^14^C dating (Supplementary Table 1)] indicated that the laminated sediments of the core are most likely annual^10,11,19^ (Supplementary Fig. 4). The pollen record from the upper 271 cm (~−50–5350 cal yr B.P., cal yr B.P.: before 1950 AD) of this core was published by Xu et al.^11^. In this study, we extend the study interval from ~−50 cal yr B.P. to ~9210 cal yr B.P.^10^.

We also compiled a dataset of 627 ^14^C dates from 98 archeological sites in NE China, bounded by the Chinese Loess Plateau-North China Plain in the south and the Inner Mongolian Plateau-Greater Khingan Mountains in the west. 584 of the dates were extracted from the database for China (*N* = 4656)^1^ and the others were from recent publications (Methods) (Fig. 1). 550 averaged ^14^C dates were calibrated using standard methods and software, following rigorous screening and elimination of dates from unsuitable materials such as shells, soils, and materials from temples, pagodas and canoes^1^. The resulting summed probability values were then plotted along the abscissa in decadal intervals using the Sum function in the CALIB 7.0.4 program^23^ and the IntCal13 calibration curve^24^. The major peaks and troughs in these summed probability distributions are regarded as evidence of increased and decreased human activity, respectively, with the steepness of the gradient of an increase/decrease indicating the rapidity of development and the intensity of human activity^25^.

^14^C dates for the above-mentioned six prehistoric cultures in NE China were assembled from the datasets (Methods). We calibrated these averaged dates (95.4% confidence level) and generated summed probability values for each culture using the Sum function in the CALIB 7.04 program^23^ and the IntCal13 calibration curve^24^. The frequency distribution values for each culture were plotted using colorized filled contours.

### ~500-yr cycle of vegetation and monsoon oscillations

**Fig. 2:**
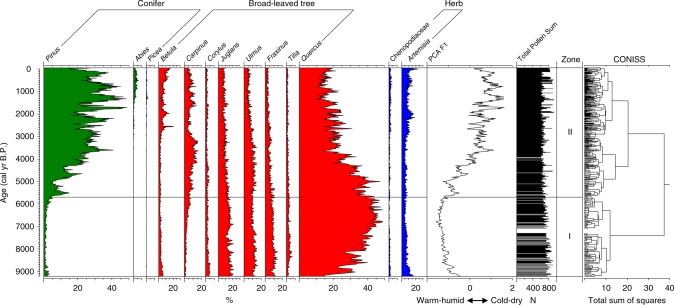
Pollen diagram of major pollen types and PCA F1 sample scores for Lake Xiaolongwan. The green, red, and blue silhouettes represent conifers, deciduous broad-leaved trees, and herbs, respectively. Positive PCA F1 sample scores represent cold-dry climatic conditions, whereas negative PCA F1 sample scores represent warm-humid conditions

The pollen record (Supplementary Data 1) from Maar Lake Xiaolongwan is illustrated in Fig. 2. The percentages of the dominant pollen types, including the elements of temperate mixed deciduous broadleaved and coniferous forest (e.g. *Pinus*, *Betula*, *Carpinus*, *Quercus* and *Ulmus*) and herb taxa (e.g. *Artemisia*), show significant changes on centennial and millennial timescales during the last 9260 yr. According to the pollen assemblages and the results of cluster analysis (CONISS)^26^, we divided the sequence into two major stages: (1) from ~9210 to 5700 cal yr B.P., broadleaved trees (e.g. *Juglans*, *Fraxinus*, *Ulmus*, and *Quercus*) are dominant, and the percentages of *Quercus* exhibit regular oscillations; and (2) after ~5700 cal yr B.P., *Pinus, Betula*, and *Artemisia* exhibit an increasing trend, and *Quercus*, *Carpinus* and *Ulmus* a decreasing trend. Note that both *Pinus* and *Quercus* exhibit anti-phased oscillations with the large amplitude of ~10–20%.

Principal components analysis (PCA) was conducted on the terrestrial pollen percentages to extract the main gradients in the vegetation. All pollen taxa with a relative abundance >2% in at least two samples were used. The first and second principal components (PCA F1 and PCA F2) have eigenvalues of 0.83 and 0.08, explaining 83% and 8% of the total variance, respectively (Supplementary Fig. 5).

The thermophilous and hygrophilous taxa, *Quercus, Ulmus*, *Juglans* and *Fraxinus*, have negative loadings on axis 1, whereas the cold-tolerant taxa, *Pinus*, *Abies*, *Betula*, and drought-tolerant taxa such as *Artemisia* and Chenopodiaceae, have positive loadings. *Quercus* is a major component of deciduous broadleaved forest, while *Pinus* is the main component of temperate mixed deciduous broadleaved and coniferous forest^27^. The anti-phased fluctuations between *Pinus* and *Quercus* thus represent changes in the relative proportions of coniferous and deciduous broadleaved trees in the forest communities^28,29^ and thus the dynamics of regional vegetation change in the Changbai Mountains^30^. Hence, PCA F1 reflects major changes in regional vegetation in the pollen source area of the lake.

The Changbai Mountains region is a typical temperate monsoon climate zone^27^. Studies of the factors controlling the distribution of montane vegetation zones indicate that the vegetation dynamics in the study region are controlled by temperature and precipitation^31–34^. Furthermore, deciduous broadleaved trees favor a warm and moist environment, while temperate mixed deciduous broadleaved and coniferous forest can tolerate cooler and drier conditions than deciduous broadleaved forest in the montane region of NE China. Therefore, PCA F1 not only reflects regional vegetation changes, but it also indicates monsoon climate changes: i.e. high *Pinus* frequencies and positive PCA F1 sample scores represent a predominance of coniferous forest and cold-dry climatic conditions, whereas high *Quercus* percentages and negative PCA F1 sample scores imply the expansion of temperate deciduous forest and warm-humid conditions.

Although our pollen record reveals a gradual, long-term, insolation-forced pattern (Supplementary Fig. 6 and Supplementary Note 2) of vegetation succession and climate change (Fig. 2), the most striking feature is that the frequencies of *Pinus* and *Quercus*, and PCA F1 sample scores, exhibit a series of multi-centennial oscillations superimposed on this general pattern (Fig. 2). As *Quercus* decreases to a minimum, *Pinus* increases to a maximum and the PCA F1 sample scores increase. The interval between two *Quercus* minima (and two peaks in PCA F1 sample scores and in *Pinus*) is ~500-yr.

**Fig. 3:**
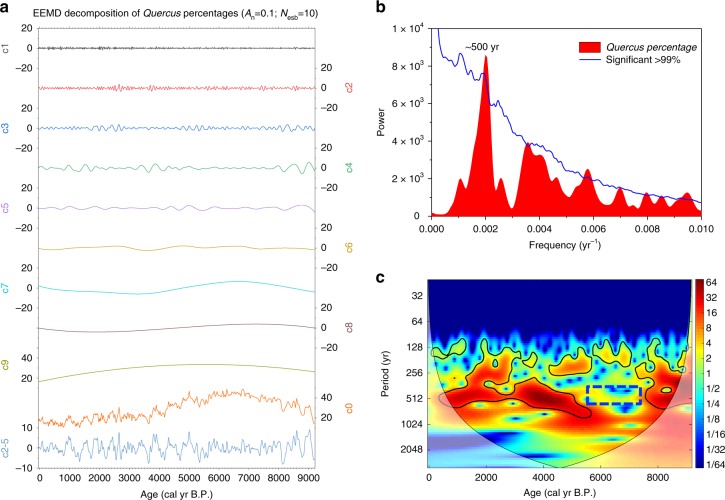
Results of time-series analysis of the *Quercus* percentages for NE China. **a** EEMD^35,36^: white noise (*A*_n_) of 0.1 and the component number (*N*_esb_) of $10 \approx \left( {{\mathrm{{Log}}}_2^{922} + 1} \right)$ are used for the first EEMD component. Component c0 represents the original *Quercus* percentages. To remove high-frequency fluctuations and orbital and millennial trends, a new detrended time series (c2-5) was generated by summing components c2-c5 from the first decomposition (c0). **b** Results of univariate spectral analysis^37^ of the *Quercus* percentages time-series over the past 9260 yr. **c** Wavelet power spectrum^38^ of *Quercus* percentages. The 95% confidence level is outlined in black. The blue dotted box indicates weak oscillations of ~500-yr cyclicity during the period of ~7500 to 5700 cal yr B.P

We performed Hilbert–Huang Transform (HHT) Ensemble empirical mode decomposition (EEMD)^35,36^ on the *Quercus* percentages and PCA F1 sample scores to remove the long-term trend and decadal-scale noise (Fig. 3a and Supplementary Fig. 7a). The c2-c5 summed components (Fig. 3a: c2-5 and Supplementary Fig. 7a: c2-5) of *Quercus* percentages and PCA F1 sample scores show clear oscillations. Redfit spectral^37^, wavelet^38^ and filter analysis were applied to determine the periodicities and periodic stabilities. The spectra of the detrended (c2-5) *Quercus* percentages and PCA F1 sample scores reveal peaks at an ~500-yr period, significant at the 99% confidence level (Fig. 3b and Supplementary Fig. 7). The results of the wavelet analysis show that the ~500-yr quasi-periodic oscillations in *Quercus* percentages and PCA F1 sample scores are near-stable and dominant throughout the last 9260 yr, except for a period of weak representation from ~7500 to 6000 cal yr B.P. (Fig. 3c and Supplementary Fig. 7c). The band-pass filtering results of *Quercus* percentages reveals a total of 13 ~500-yr cold-dry and warm-humid oscillations from ~9210 to 2500 cal yr B.P. (Supplementary Fig. 8). The warm-humid climatic optima occur at ~8970, ~8470, ~7970, ~7420, ~6830, ~6350, ~5940, ~5450, ~4980, ~4390, ~3900, ~3400, ~2810 cal yr B.P. (Supplementary Fig. 8). Pollen records from Maar Lake Sihailongwan^34^ (20 km from Maar Lake Xiaolongwan) and Jeju Island in the Yellow Sea^5^, and the speleothems record from Heshang cave^39^ in South China, also show ~500-yr summer monsoon oscillations during the mid-late Holocene (Supplementary Fig. 9 and Supplementary Note 3). Our record of long-chain n-alkanes δ^13^C_27–31_, an effective humidity proxy from the same core, shows a strong ~500-yr periodicity^10^ (Supplementary Fig. 10 and Supplementary Note 3). In addition, a 200-yr precipitation record from an adjacent weather station in Changchun City^40^ maps convincingly on the final stage of the pollen-based ~500-yr monsoon cycles (Supplementary Fig. 11), supporting the conclusion that our pollen record is a monsoonal climate proxy.

Moreover, the temporal framework demonstrates that a northward advance of the EASM rain belt could have led to warm and humid conditions favorable for *Quercus*, whereas a southward retreat of the EASM rain belt could have caused a cold and arid environment unfavorable for *Quercus* in NE Asia^5,41^. Thus, these studies all support the conclusion that our pollen record clearly shows that temporal vegetation and environmental changes were driven by ~ 500-yr periodic EASM oscillations. However, the EASM was the strongest during the period of 7500–5500 cal yr B.P., and such climatic conditions may have suppressed the signal of ~ 500-yr monsoon cyclicity in the study area.

### ~ 500-yr cyclical change in human activity

**Fig. 4:**
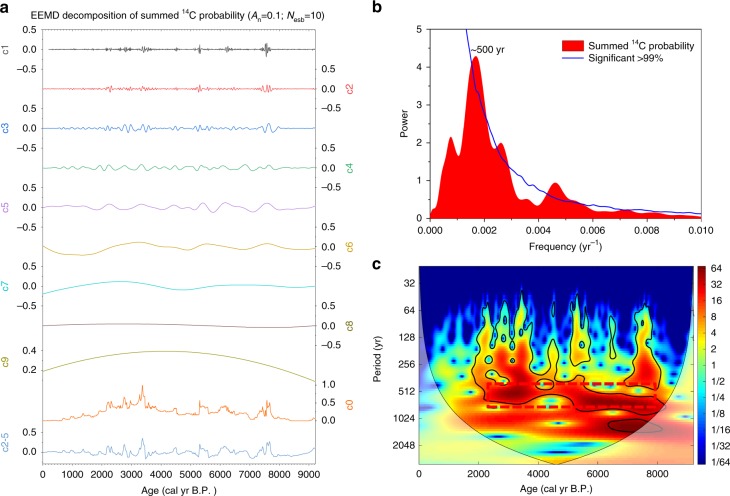
Results of time-series analysis of the SCP record for NE China. (**a**) EEMD^35,36^: white noise (A_n_) of 0.1 and the component number (N_esb_) of $10 \approx \left( {Log_2^{922} + 1} \right)$ are used for the first EEMD component. Component c0 represents the original SCP data. To remove high-frequency fluctuations and orbital and millennial trends, a new detrended time series (c2-5) was formed by summing components c2-c5 from the first decomposition (c0). (**b**) Results of univariate spectral analysis^37^ of the SCP time-series over the past 8000 yr. (**c**) Wavelet power spectrum^38^ of SCP. The 95% confidence level is outlined in black. The red dotted box indicates the strong oscillations of ~ 500-yr cycles during the period of ~ 8000 to 2000 cal yr B.P

The summed ^14^C probability (SCP) record exhibits lower values ( < 0.286) and weak variability ( < 0.184) before 8000 cal yr B.P. and after 2000 cal yr B.P. (Fig. 4a: c0, Supplementary Fig. 12 & Supplementary Note 4). This may be due to the relative paucity of archaeological sites and dates before 8000 cal yr B.P. and the increased availability of historical documentary evidence after 2500 cal yr B.P.. The SCP remains high (from 0.042 to 0.998), with notable fluctuations (from 0.167 to 0.648), from 8000 to 2000 cal yr B.P. (Fig. 4a: c0); the peak values are at ~ 7570, ~ 6770, ~ 6240, ~ 5440, ~ 4930, ~ 4420, ~ 3820, ~ 3300 and ~ 2800 cal yr B.P. (Supplementary Fig. 13b).

The SCP curve for NE China was detrended using HHT EEMD^35,36^ (Fig. 4a). The c2-c5 summed components (Fig. 4a: c2-5) of EEMD and wavelet analysis results indicate a strong ~500-yr oscillation from 8000 to 2500 cal yr B.P. (Fig. 4a, c). Redfit spectral analysis also shows a dominant ~500-yr cyclicity during this interval (Fig. 4b). Thus, the consistency of the characteristics of the time-series analysis results of the SCP indicates a dominant ~500-yr cycle of the intensity of human activity in NE China (Fig. 4).

### Synchronous ~500-yr cyclic changes in EASM and human activity

**Fig. 5:**
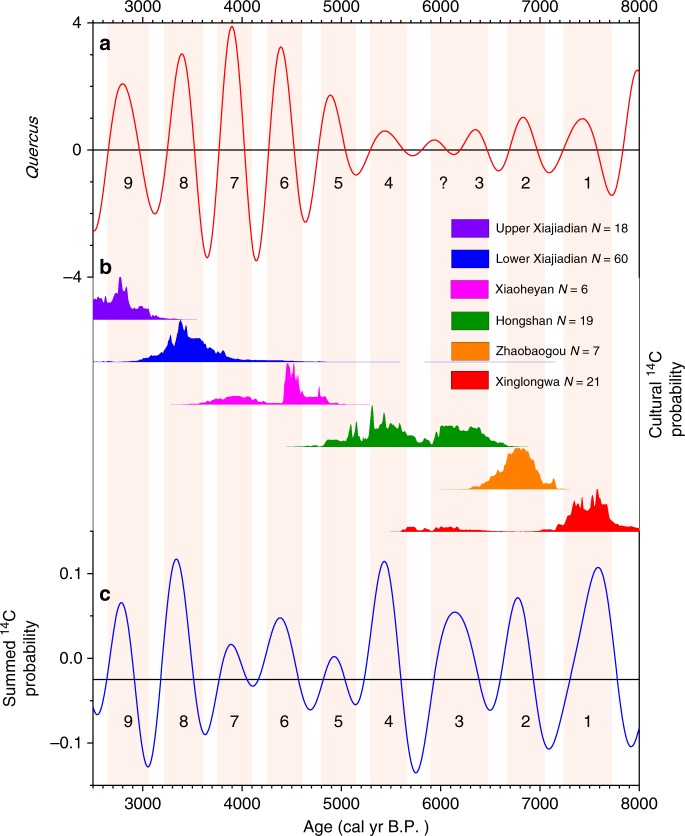
Comparison of the detrended *Quercus* record (EASM proxy) with detrended ^14^C probability. **a** 400–600-yr band-pass filter of the detrended *Quercus* record. The central frequencies and bandwidths of the *Quercus* percentages (EASM, c2-c5 component after HHT analysis) filters are 0.020 yr^−1^ (500-yr period), 0.017 yr^−1^ (600-yr period), and 0.025 yr^−1^ (400-yr period), respectively. **b** Density histogram of cultural ^14^C probability from prehistorical archeological sites; the ^14^C-dated sites belong to the Xinglongwa, Zhaobaogou, Hongshan, Xiaoheyan, Lower Xiajiadian and Upper Xiajiadian cultures. **c** 400–1250-yr band-pass filter of the detrended SCP record. The central frequencies and bandwidths of the SCP (c2-c5 component after HHT analysis) filters are 0.020 yr^−1^ (500-yr period), 0.008 yr^−1^ (1250-yr period), and 0.025 yr^−1^ (400-yr period), respectively. The two proxies (EASM and SCP) show nine ~500-yr cycles. The ~500-yr cycle of SCP is almost in-phase with ~500-yr oscillations of EASM. The density histogram of SCP likely reflects the flourishing and decline of the Xinglongwa, Zhaobaogou, Hongshan, Xiaoheyan and Lower Xiajiadian and Upper Xiajiadian cultures. The light-red vertical bars show nine periods of intense human activity and flourishing of prehistoric culture

Figure 5 displays the ~500-yr band pass filter results of the detrended *Quercus* percentages (summed components c2-c5), and the detrended SCP (summed components c2-c5), together with the frequency distribution of prehistorical cultural ^14^C probability in NE China. The ~500-yr cycle of changing human activity (Fig. 5c) is almost in-phase with (cycles 1-2 and 4-9) or lags by several decades (cycle 3) the ~500-yr cycle of warm-humid/cold-dry climatic oscillations (Fig. 5a, Supplementary Fig. 13). In addition, the timing of the ~500-yr cycles of the intensity of human activity (SCP) and the flourishing/decline of prehistoric cultures (Fig. 5b, c) show similar temporal patterns. Thus, the cyclicity of human activity in NE China during the Holocene was almost synchronous and highly coherent with the EASM fluctuations.

Based on the ^14^C probability record from the archeological sites in NE China, the intervals during when prehistoric cultures flourished are: ~7700–7200 cal yr B.P. (Xinglongwa); ~7000–6700 cal yr B.P. (Zhaobaogou); ~6500–5900, ~5700–5300 and ~5100–4800 cal yr B.P. (Hongshan); ~4600–4300 and ~4100–3700 cal yr B.P. (Xiaoheyan) and ~3600–3200 cal yr B.P. (Lower Xiajiadian) and ~3000–2600 cal yr B.P. (Upper Xiajiadian) (Fig. 5b). In general, the phases of prosperous prehistoric cultures coincide with the warm-humid phases of the ~500-yr monsoon climate cycles (Fig. 5a, b).

However, the amplitudes of the ~500-yr periodicities in the *Quercus* record do not correspond exactly to either the periodic changes of SCP or the frequency distribution of cultural ^14^C probability (Fig. 5a, c and Supplementary Fig. 13). The most obvious difference is that the amplitudes of cycles 5 and 7 are relatively large in the *Quercus* record and small in the SCP record. In addition, the amplitudes of cycles 3 and 4 are small in the *Quercus* record and large in the SCP record. Moreover, from ~6500 to 5300 cal yr B.P., between cycles 3 and 4, three relatively weak warm-humid peaks are indicated by the *Quercus* record (Fig. 5a), but the record of human activity shows only two peaks (Fig. 5b, c). Interestingly, this period witnessed the most significant social development in the region, known as the Hongshan culture; thus, the relationship between climatic conditions and human activities needs to be investigated in greater detail.

The economic foundations of these cultures were associated with the gradual transition from a broad-spectrum subsistence strategy based on millet farming, pig husbandry, and hunting-gathering wild foods, to an intensified millet agriculture with pig domestication, and then to agro-pastoralist subsistence strategies, which were all highly dependent on a warm environment and adequate precipitation^13,15,42^. Warm and humid conditions favored the increased productivity of wild plants, cultivated crops, wild animals and livestock^1,16^. An abundant food supply would have promoted the growth of population, an expansion of the area of occupation, and overall prosperity^1,16^.

The sites of the six cultures are distributed in the present EASM northern marginal region^43,44^. Among the six prehistoric cultures, the early Xinglongwa and Zhaobaogou, which were characterized by a subsistence economy dominated by hunting-gathering with a minor farming component, are closely correlated with 500-yr climatic cyclicity in the early Holocene when the EASM began to strengthen.

At ~6500–5300 cal yr B.P. (cycles 3 and 4), the warmest and wettest period of the Holocene, the EASM marginal region migrated at least 150–200 km northwards^22,41^. This may have suppressed the signal of the 500-yr monsoon cycle in this area. The prosperity of the Hongshan culture during the middle Holocene generally corresponds to the occurrence of warm and humid conditions (Supplementary Fig. 14). However, during ~6500–4800 cal yr B.P., two strong peaks and a weak peak in human activity are evident (Fig. 5b, c: SCP 3, 4 and 5), described here as early, middle, and late Hongshan phases, respectively. The ^14^C dates from the large ritual centers at Niuheliang and Dongshanzui fall within the middle phase (~5800–5300 cal yr B.P., Supplementary Fig. 15), corresponding to the warmest and wettest period indicated by the trend of the *Quercus* record (Supplementary Fig. 14). However, the ^14^C dates of the late phase (~5100–4800 cal yr B.P.) are all derived from small sites which exhibit no clear evidence of large-scale ritual architecture^15^.

This pattern suggests that the rise and fall of the Hongshan complex society may have been related to a series of societal responses to environmental fluctuations, including the intensification of land use for agriculture, the construction of monumental architecture for religious ceremonies, and the development of elite ritual powers. Hongshan ritual activity (Supplementary Fig. 16) reached a peak during the middle phase (~5800–5300 cal yr B.P.), represented by Niuheliang and Dongshanzui (Supplementary Fig. 15), and ended at about 5300 cal yr B.P. However, the termination of elite activities at large ritual centers does not imply a total collapse of the Hongshan culture as previously thought; instead, it marked the disappearance of elite power with associated material symbols. The middle phase was followed by a period of several hundred years of continuous human occupation in small residential settlements during the late phase.

The late phase ended at around 4800 cal yr B.P., which coincides with an episode of climatic deterioration caused by a superposition of ~500-yr cycles and the long-term climatic trend (Fig. 5 and Supplementary Fig. 14). If the rise and fall of the Hongshan elite was related, in part, to climatic fluctuations such as recurrent droughts, the continuous presence of the Hongshan population during the late phase indicates that the human response to a worsened climate included the rejection of the elite (who were seemingly unable to deliver favorable weather as reliably as before), the modification of social organization, and changing cultural values^15^. A similar scenario occurred in the case of the Classic Maya collapses in Mesoamerica^45,46^.

However, as most archaeologists believe, prehistoric cultures possessed social resilience to climate change^47^. The Hongshan people’s strategy for coping with the deteriorating climate and environment included changes in settlement location and religion practices^15^. The late phase of the Niuheliang, Dongshanzui, Caomaoshan and Wudaowan (Supplementary Fig. 15) was characterized by small, sparse and scattered sites with few examples of ritual and public architecture^15^. On the other hand, the collapse of a prehistoric culture may not represent a complete collapse, since the succeeding culture can inherit the older one to a certain extent. Archeological evidence shows that the Lower Xiajiadian culture adopted several aspects of the Hongshan culture^43^. For example, many attributes of the ritual sites and jade and ceramic manufacturing technology of the Lower Xiajiadian culture are derived from the Hongshan culture (Supplementary Fig. 16)^43^.

### ~500-yr ENSO cycle linkages between EASM and human activity

NE China is a marginal area affected by the EAM^11,22^, and its ecological environment is very sensitive to monsoon advance and retreat^11^. Among the various factors affecting the change of the EAM, ENSO has the greatest impact on NE China on the inter-annual and inter-decade scale^48^. Moreover, ENSO variance exhibits ~500-yr cyclic change during the entire Holocene (Supplementary Fig. 17) and regulates EAM changes on the centennial scale^5,39,49,50^. ENSO can drive the movement of the rainbelt caused by intensifying the interaction between the western Pacific anticyclone and dipolar sea surface temperature anomalies in the Indo-Pacific warm pool and the Northwest Pacific^48^. It could also weaken the western Pacific subtropical high and limit the extension of the rainbelt to NE China^51^. During the El Niño-like phases there was a weakening of the EASM and southward migration of the rain belt, resulting in reduced precipitation in NE China; and in La Niña-like (or normal) phases, a strengthening of the EASM triggered the northward movement of the rain belt, resulting in more rainfall in NE China^5,11,39,52^.

The band pass filter and cross wavelet transform (XWT) results show that the ~500-yr cycle of ENSO and EASM exhibits large amplitudes and nearly in-phase oscillations from ~8000 to 6500 cal yr B.P. and from ~5500 to 2500 cal yr B.P. They also show that from ~6500 to 5500 cal yr B.P., the ~500-yr cycle of ENSO and EASM exhibits weak amplitudes and a large offset, with ENSO leading EASM^39,49^ (Supplementary Fig. 18a & b). The ~500-yr cycles of the EASM and SCP are nearly in phase from ~8000 to 6500 cal yr B.P. and from ~5500 to 2500 cal yr B.P., but they are out of phase from ~6500 to 5500 cal yr B.P. (Supplementary Fig. 18a, c). The out of phase relationship between SCP and EASM from ~6500 to 5500 cal yr B.P. may have been related to social resilience^15^. This phenomenon implies a close relationship between ENSO variability and the ~500-yr cycle of EASM and SCP variations^5,11,34^. In addition, we have previously demonstrated that the ~500-yr climate cyclicity in the EAM region was affected by the Arctic Oscillation//North Atlantic Oscillation (AO/NAO) in the high-latitude climate system^11^. Thus, the ~500-yr cyclicity in cultural prosperity and development and EASM changes in NE China may be related to interactions of the high and low-latitude components of the climate system^51^. The ~500-yr cycles are both significant components of ENSO in the low-latitude region^5,39^ and of AO/NAO^53,54^ in the high-latitude region, and their changes may be related to fluctuations in solar activity.

It should be noted that the ~500-yr cycles are superimposed on a long-term trend. The general climatic trend of the Holocene contributed to the pattern of stability and prosperity of prehistoric cultures, while the ~500-yr periodicities affected the intensity of prehistoric human activity on the centennial scale.

## Discussion

In summary, our results reveal an ~500-yr cyclicity in variations of EASM monsoon climate and human activity in NE China during the last 9260 yr, with a near synchronous relationship between them. Monsoon-induced ~500-yr cyclic changes in ecosystems in NE China evidently had major impacts on human activity and cultural change. A strengthened EASM would have promoted increased plant and animal productivity and hence material and cultural prosperity. We have reliably identified a closely coupled relationship between changes in human culture and in climate and environment, using a record of decadal resolution. We have also demonstrated that the coupled changes were periodic and on a centennial scale, in contrast to prior studies which focused on millennial-scale events. Overall, we are confident that our results constitute robust evidence that the ~500-yr cyclic variations in human activity and climate change were influenced by ENSO and AO/NAO variability.

Nevertheless, the relationship was not always straightforward, since humans responded to the superimposed climatic changes in different ways. In the case of the early-mid Hongshan culture, relatively weak ~500-yr cyclic monsoon oscillations, corresponding to the strongest period of the mid Holocene monsoon, were associated with very high levels of human activity. While the emergence of unparalleled ritual power acquired by the Hongshan elite may have been a human spiritual response to accommodate sub-optimal environmental conditions, the subsequent decline of elite culture likely reflected a different social strategy used by ordinary people to cope with worsened climatic fluctuations. Overall, our findings reveal a close correspondence between ecosystem change and the archeological record in NE China, and they provide insights into the long-term interrelationship between cyclical centennial-scale climate change and prehistoric human activities in the EAM region.

## Methods

### Varve counting

The sediment cores were cut into slabs of 6.5-cm length, with a 1.5-cm overlap, and were then shock-frozen, vacuum-dried and impregnated with epoxy resin prior to prepare thin sections. Varves were identified and counted from thin sections at different magnifications (×4, ×6.5, ×20) using a Leitz optical microscope.

### ^137^Cs and ^210^Pb dating

The upper 19 cm of the core were sampled at a 1-cm interval for ^137^Cs and ^210^Pb dating (Supplementary Fig. 3). Vacuum-dried samples were measured for radionuclides in a well-type germanium detector (EGPC 100P-15R). Each sample was packed in a 15-mm polyethylene tube and stored for 3 weeks in sealed containers to allow for radioactive equilibration, and then counted for 48 h. ^210^Pb was determined via its energy emission at 46.5 keV. ^226^Ra was determined by gamma spectrometry, based on measurements of ^214^Pb (241.9, 295.2 and 351.9 keV) and ^214^Bi (609.3 keV) photo peaks. ^137^Cs was measured via its emissions at 662 keV. The unsupported ^210^Pb activity (^210^Pb_unsup_), obtained by subtracting ^226^Ra, was used to estimate average linear sedimentation rates for the core. Radiometric dates were calculated from the ^210^Pb and ^137^Cs records using the CRS model^55^.

### Radiocarbon dating

The radiocarbon chronology for the upper 387 cm of the core is reported elsewhere^10^. The reservoir correction factor is 212 yr for the uppermost 20 cm of the core. Corrected ^14^C ages with 2σ-range were obtained using the IntCal13 data set^24^ from the CALIB 7.04 program^23^.

### Pollen preparation procedures

Samples of weight 0.3 ± 0.05 g at 1-cm intervals (average resolution of 25-yr) were prepared for pollen analysis. This interval was selected for thin section preparation for varve counting to ensure the best temporal correlation between the pollen and varve data. All samples were treated with KOH, HCl, HF and a hot acetolysis mixture^56^. *Lycopodium* spores (27637 per tablet) were added to each sample to calculate pollen concentrations. Sample residues were suspended in glycerine and analyzed using a Leica DM750 microscope at ×400 magnification. Pollen preservation was excellent and the pollen concentration was high. An average of 701 (range 585–949) terrestrial pollen grains was counted for each sample (Fig. 2 and Supplementary Fig. 19). A total of 378 pollen samples were analyzed, with an average temporal resolution of ~25 yr. TILIA 2.0.41 (ref. ^26^) was used to calculate pollen percentages and to plot the pollen diagram. Pollen percentages were calculated on the sum of total terrestrial pollen, excluding the pollen of aquatic plants and fern spores.

### Radiocarbon dates from archeological sites in NE China

We screened uncalibrated ^14^C dates using the criteria described by Maher et al.^57^ and Wang et al.^1^. The following dates were eliminated: (1) dates with high error bars (1σ standard deviation >400 ^14^C yr); (2) dates from shells, soils, unknown materials or other materials considered inappropriate for dating; and (3) dates derived from sites or materials that had weak associations with human occupation or settlement, such as ancient temples, pagodas or canoes. In cases where dates were derived from several sample materials (e.g. charcoal and shell, or charcoal and charred millet seed), both obtained in the same context, the most reliable dating material was chosen. The remainder of the database contained 550 dates, have errors of 84.1 yrs (see Supplementary Data 2). We used the R_Combine command within OxCal v4.2.3 to combine the redundant dates. This approach has two advantages: (1) reducing the standard deviation and increasing the accuracy of each site’s temporal assignments; and (2) reducing sampling bias created by sites/phases with numerous radiocarbon dates during statistical analyses^58,59^. We calibrated averaged dates (95.4% confidence) and generated summed probability values for the regions of NE China using the Sum function in the CALIB 7.04 program^23^ and the IntCal13 calibration curve^24^: (1) the individual calibrated probability distribution is generated using the calibration command; and (2) summed probability values are calculated by the sum probabilities command. We also applied the empirical model proposed by Surovell et al.^60^ to correct for taphonomic bias, since it is assumed that older dates may be underestimates due to natural destructive processes^60^. After correction, the data were standardized (normalized) as follows: *X*_*i*_/*X*_max_, where *X*_*i*_ is each single value and *X*_max_ is the maximum value in the series.

### Reporting summary

Further information on research design is available in the Nature Research Reporting Summary linked to this article.

## Supplementary information

Supplementary Information

Reporting Summary

Description of Additional Supplementary Files

Supplementary Data 1

Supplementary Data 2

## Data Availability

All data generated or analyzed during this study are included in this published article (and its supplementary information files).
