# Supplementary material for: Synchronous 500-year oscillations of monsoon climate and human activity in Northeast Asia
Source: Nat Commun. 2019 Sep 11;10:4105. doi: 10.1038/s41467-019-12138-0 (PMC6739325; doi:10.1038/s41467-019-12138-0)
Supplement: Supplementary file 1 — Supplementary Information [file 41467_2019_12138_MOESM1_ESM.pdf]

SUPPLEMENTARY INFORMATION

Synchronous 500-year oscillations of monsoon climate and human activity in Northeast Asia

Xu et al.

This file includes:

Supplementary Notes

Supplementary Note 1: Background information about Maar Lake Xiaolongwan

Supplementary Note 2: Insolation forcing of vegetation changes in NE China

Supplementary Note 3: ~500-year monsoonal climate cycles recorded in East Asia

Supplementary Note 4: Radiocarbon (^{14}C) dates from archeological sites in NE China

Supplementary Figures

Supplementary Figures 1 to 19

Supplementary Table

Supplementary Table 1

Supplementary References

Supplementary Notes

Supplementary Note 1: Background information about Maar Lake Xiaolongwan

The area of Maar Lake Xiaolongwan is 0.079 km^2 and the maximum depth is 15 m; there are no inflowing or outflowing rivers [1] (Supplementary Fig. 1). The lake is dystrophic and humic, with brown-colored water containing a high concentration of organic matter and with a low pH [2]. The lake has seasonal dinoflagellate blooms and the sediments are annually laminated [3]. We conclude that the pollen content of the sediments is supplied by air, rainout, and surface runoff, with no contributions and disturbance from inflows/outflows. The unambiguous pollen source and excellent depositional and preservation conditions potentially guarantee the reliability of the pollen record [4].

Supplementary Note 2: Insolation forcing of the main trend of vegetation change in NE China

The main trend of *Quercus* percentages in the pollen record tracks changes in 65ºN JJA insolation, and thus the long-term trend of the pollen record from Maar Lake Xiaolongwan was mainly forced by changes in Northern Hemisphere insolation during the Holocene (Supplementary Fig. 6).

Supplementary Note 3: ~500-year monsoonal climate cycles recorded in East Asia

Records of ~500-yr monsoonal climate periodicity in East Asia. The pollen record from Maar Lake Sihailongwan in NE China[5], a pollen record from a crater swamp in Jeju Island in the Yellow Sea [6], and a speleothem record from Heshang cave in South China[7], all provide evidence of ~500-yr monsoonal climatic cyclicity in East Asia. The confidence levels of the ~500-yr periodicities are all higher than 90% (Supplementary Fig. 9). Our pollen record from annually-laminated Lake Xiaolongwan, with cross validated dates and a high resolution (decadal scale), the ~500-yr monsoonal climate cycles is significant at the 99% confident level (Fig. 3). Overall, the ~500-yr periodicity recorded by our pollen record is clearer and more reliable than that of other records from East Asia (Fig. 3 & Supplementary Fig. 9).

~500-yr climate cycles recorded by carbon isotopes of long-chain n-alkanes.
Multi-proxies and their mutual corroboration can provide more comprehensive and reliable information about monsoonal climate changes than an individual proxy. Thus, other independent proxies are needed to assess the reliability of monsoon climate change revealed

by our pollen record. The stable carbon isotope composition of long-chain n-alkanes ($\delta^{13}C_{27-31}$), an effective humidity proxy[8] , was studied in the same core from Maar Lake Xiaolongwan. The $\delta^{13}C_{27-31}$ record shows strong ~500-yr oscillations (significance >95%), and in addition other periodicities (e.g. ~200-yr) exceed the 95% confidence level. Notably the long-chain n-alkanes proxy is based on multiple sources of terrestrial higher plant material [8] (Supplementary Fig. 10).

Supplementary Note 4: Radiocarbon (^{14}C) dates from archeological sites in Northeast China

Previous studies showed that human occupation leads to the production and deposition of increased quantities of cultural carbon [9-12]. Good preservation of the deposited carbon results in the increased recovery of carbon by archaeologists; moreover, extensive archaeological investigations will result in the increased recovery of the carbon and hence of dating samples [9]. Thus, the abundance of radiocarbon dates is a potential proxy of the human population of a region during a given period [9]. However, archeologists are faced with the issue of the validity of using “dates as data” in the case of archeological ^{14}C dates. Using “dates as data” is the common name for the practice of using ^{14}C dates as a form of data which is assumed to reflect spatiotemporal changes in paleodemography. However, it may be difficult to test the validity of this assumption [13]; moreover, it includes biases and errors associated with archaeological studies, such as the taphonomic loss of samples and varying sample size [12], as well as issues associated with the analysis of the databases of the studied sites. These problems can be attributed to quality issues, sample size, sampling bias, temporal bias, taphonomic issues and human factors [14, 15]. Although archeological radiocarbon dates are affected by the foregoing issues, we attempted to minimize the bias using the following statistical approach [12, 13, 16].

(1) As Williams [12] pointed out, a sufficiently large regional sample set from a large assembly of sites can be viewed as a quasi-random sample set without site and period level biases. Thus, it can be assumed to be statistically reliable for a robust summed probability distribution reflecting an actual trend in population. Therefore, it is very important for archeologists to collect adequate samples to reduce sampling bias. The working assumption of summed probability analysis is that a sufficiently large sample of radiocarbon dates from a large enough area will constitute legitimate evidence for indicating population fluctuations[10-12].

In this study, we re-selected ^{14}C dates from the integrated database of archeological radiocarbon dates for China [17]. The 4656 ^{14}C dates in the database were mainly obtained from published archeological ^{14}C determination datasets, reports, review papers, and completed research dissertations. We assembled 627 archeological ^{14}C dates to build a dataset for NE China, among which 584 dates were extracted from the database for China [17] and an additional 43 dates were from new publications. These dates from NE China (~1.52 million km^2) exceed the minimum size of 500 samples for a large region (~7.69 million km^2) (65 samples in an area of 1 million km^2) suggested by Williams[12], and therefore they can mitigate site- and period- level biases. Over 95% (n=598) of the dates have errors of 400 years or less. Following Williams' [12] suggestion, we report that the mean standard deviation (△T) for the entire sample set was 123.1 years. Relevant information about the dating methods and materials used in the database, which are significant for evaluating dating reliability, are summarized (Supplementary Data 2).

(2) Although all published archeological radiocarbon dates were included in the database at the time of writing, we chose to exclude 77 ^{14}C dates from our analysis. We screened uncalibrated ^{14}C dates using the criteria described by Maher et al. [18] and Wang et al.[17]. Dates with the following attributes were excluded: (i) Dates with large error bars (1σ standard deviation >400 ^{14}C year); (ii) dates from shells, soils, unknown materials or other materials considered inappropriate for dating; and (iii) those dates derived from sites or materials that had weak associations with human occupation or settlement, such as ancient temples, pagodas or canoes. In cases where dates were derived from several sample materials (e.g. charcoal and shell, or charcoal and charred millet seed), both obtained in the same context, the most reliable dating material was chosen. The resulting database consisted of 550 dates, which still exceeds Williams' [12] suggested a minimum sample size of 500, with errors of 84.1 years (Supplementary Data 2).

(3) After screening inappropriate data, there are still 7 large-size archeological sites containing 10 or more dates. The large scale of the archeological sites may introduce a certain amount of redundancy among the dates [17], and therefore we used the R_Combine command within OxCal v4.2.3 to combine the redundant dates. This approach has two advantages: (i) it reduces the standard deviation and increases the accuracy of each site's temporal assignment; (ii) it reduces sampling bias created by sites/phases with numerous radiocarbon dates during statistical analyses [19, 20]. If one date is very similar to the others at the same archeological site then the method used combines similar dates to produce a new

date [19, 20]. This procedure reduces the weighting of sites/phases with many dates: for example, the number of dates from Xinglongwa site is reduced from 17 to 8 and its frequency in the total sample set is reduced from 3% to 1.4% (see Supplementary Data 2).

With the aid of the foregoing strategy, it seems reasonable to assume that we can mitigate the effect of different biases in the data, so that the resulting summed probability distributions can provide us a valid estimate of changes in prehistoric demography.

All the ^{14}C dates were calibrated using standard methods and software. They are reported in years before present (B.P., before present=1950 C.E.) and are based on the Libby half-life of 5568 yr with 1σ standard deviation. Calibration was conducted using the OxCal 4.2.3 program [21] and IntCal13 curve [22] with ranges expressed both at the 1σ (68.2%) and 2σ (95.4%) confidence levels. All calibrated ages reported are referenced as "cal yr B.P.". We calibrated averaged dates (95.4% confidence) and generated summed probability values for the regions of NE China using the Sum function in the CALIB 7.04 program and the IntCal13 calibration curve [22, 23]. We also applied the empirical model proposed by Surovell et al. [9] to correct for taphonomic bias, since it is assumed that older dates may be underestimates due to natural destructive processes [9]. After correction, the data were standardized (normalized) as follows: X_i/X_{max}, where X_i is each single value and X_{max} is the maximum value in the series.

The use of normalized or un-normalized calibrations does affect the shape of the summed probability distributions (SCP); however, it has no influence on the shape of a single calibrated probability distribution [24]. We also conducted the calibration using normalized and un-normalized ^{14}C dates with the rcarbon packages in R software. As a result, there are no shape differences between the CALIB and rcarbon results in the normalized calibrations (Supplementary Fig. 12). The shape of the SCP using normalized and un-normalized dates are slightly different (Supplementary Fig. 12); however, these differences only affect the long-term trend (>~1000 yr), and not the amplitude and phase of ~500-yr cycles. Thus, it is possible to obtain robust normalized calibrations.

The ^{14}C dates of Neolithic cultures (Xinglongwa, Zhaobaogou, Hongshan, Xiaoheyan) and the Bronze Age cultures (Lower Xiajiadian and Upper Xiajiadian) in NE China were assembled. We calibrated these averaged dates (95.4% confidence level) and generated summed probability values for each culture using the Sum function in CALIB 7.04 using the IntCal13 calibration curve [22, 23]. The frequency distribution values for the cultures were then plotted using colorized filled contours. The resulting summed probability values were then plotted along the abscissa in decadal intervals; the major peaks and troughs in the summed

probability distributions are regarded as evidence of increased and decreased human activity, respectively, with the steepness of the gradient reflecting the rapidity and amplitude of changes in human activity [25]. The summed probability curve and culture density histogram in NE China were reanalyzed to reconstruct spatiotemporal changes in human activity and prehistoric cultural development.

Supplementary Figures

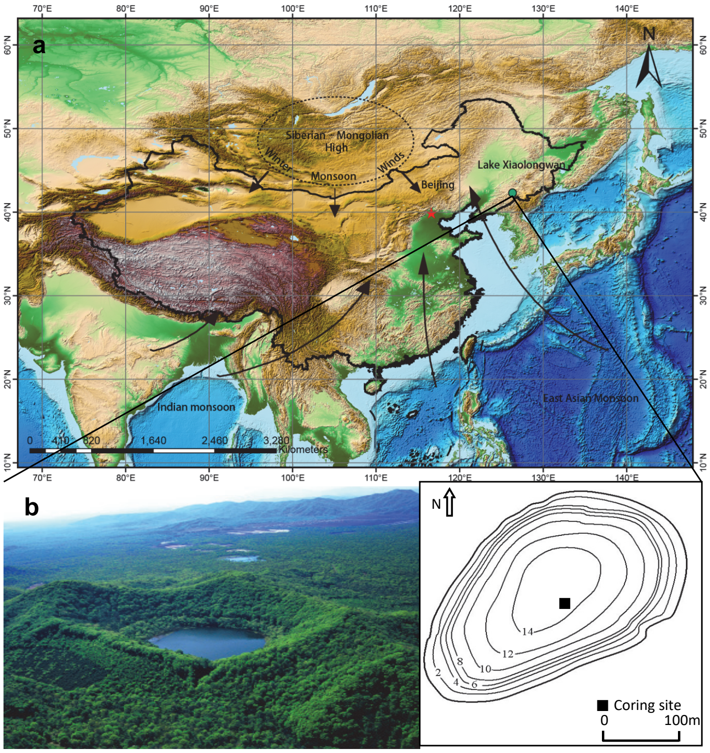

Supplementary Fig. 1. Overview of Maar Lake Xiaolongwan [4] (**a**) Location of Lake Xiaolongwan (green dot); Beijing is indicated by the red dot. Regional atmospheric circulations (black arrows) include Winter monsoon, East Asian Monsoon and Indian Monsoon. (**b**) Photo and bathymetric sketch map of Lake Xiaolongwan. Figure1a was generated using DIVA-GIS 7.5 (http://www.diva-gis.org/). The photo in Figure 1b was taken by Guoqiang Chu.

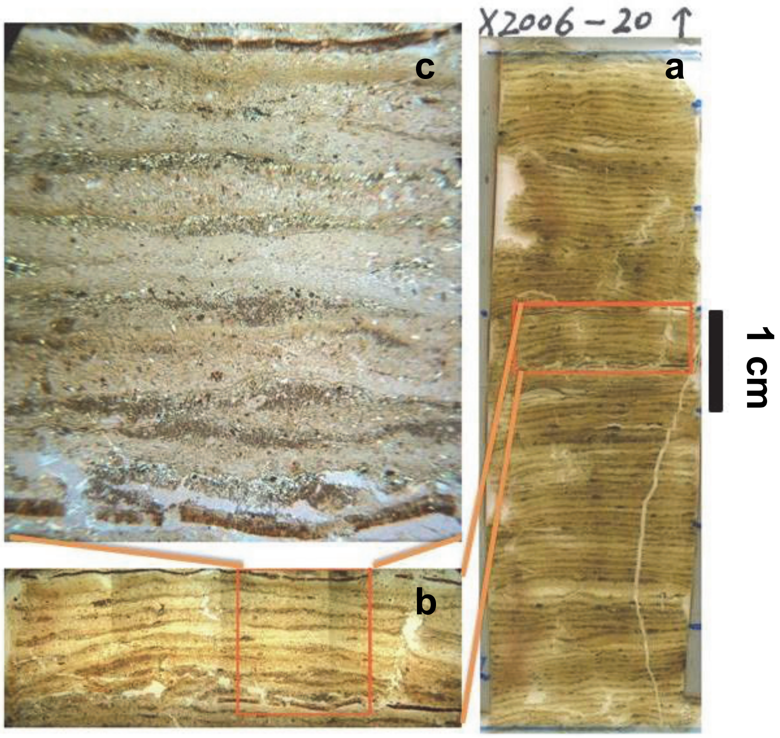

Supplementary Fig. 2. Photomicrographs showing annual laminations in the sediments of Lake Xiaolongwan. (a) Scan of thin section X2006-20. (b) Detail of laminations in the area delimited by the orange rectangle in panel a. (c) Detail of laminations in the area delimited by the orange rectangle in panel b.

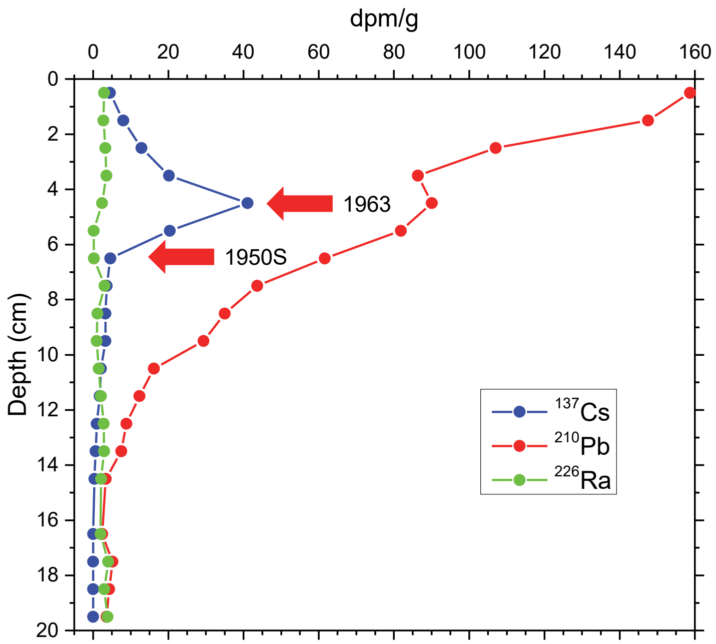

Supplementary Fig. 3. ^{137}Cs, ^{210}Pb and ^{226}Ra depth profiles for the sediment core from Lake Xiaolongwan. ^{137}Cs activity exhibits a sharp peak, indicating the absence of significant sediment mixing. The peak in ^{137}Cs (41.1 dpm/g) occurs at the depth of 4.5 cm and represents the 1963 peak in above-ground nuclear weapons testing. The ^{137}Cs value at the sediment depth of 6.5 cm is near zero (cesium horizon) and it may represent deposition at or around the onset of large-scale nuclear weapons testing in the early 1950s. The depths of the cesium peak and horizon indicate average sediment accumulation rates of 0.11 and 0.12 cm/yr, respectively. ^{210}Pb activity declines roughly exponentially with depth, indicating relatively uniform sediment accumulation during the past ~100 years, except for an increase at 4.5 cm and a decrease at 3.5 cm. ^{226}Ra activities are relatively uniform, with a mean value of 2.36 dpm/g. The ^{210}Pb chronology was calculated using the CIC model to estimate sedimentation rates.

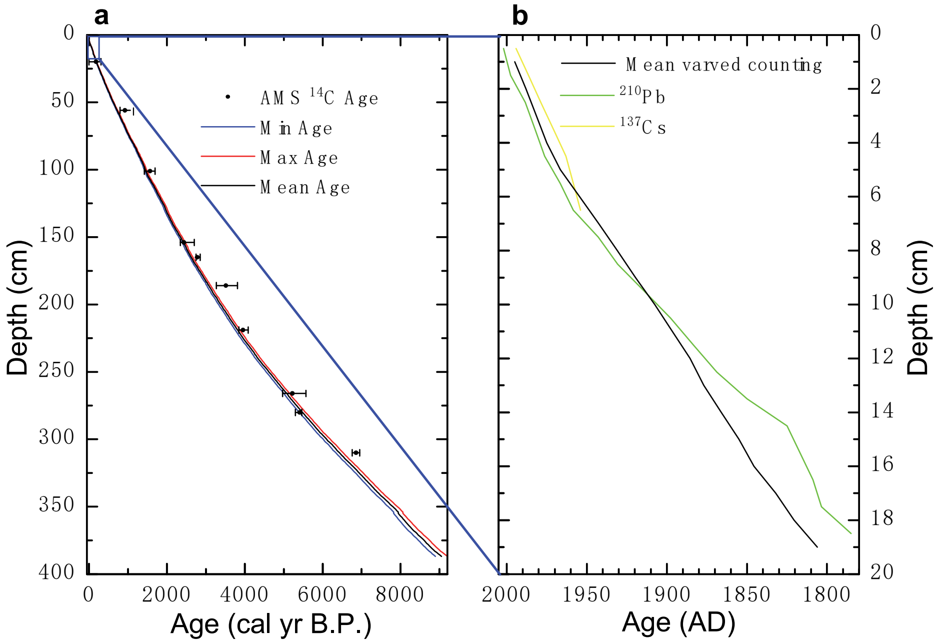

Supplementary Fig. 4. Comparison of sediment chronologies derived from ^{137}Cs, ^{210}Pb and ^{14}C dating and lamination counting for the sediment core from Lake Xiaolongwan. (**a**) Black dots: calendar AMS ^{14}C age with dating errors; blue line: minimum varved age; red line: maximum annually-laminated age; black line: mean annually-laminated age. (**b**) Green line: ^{210}Pb age; yellow line: ^{137}Cs age.

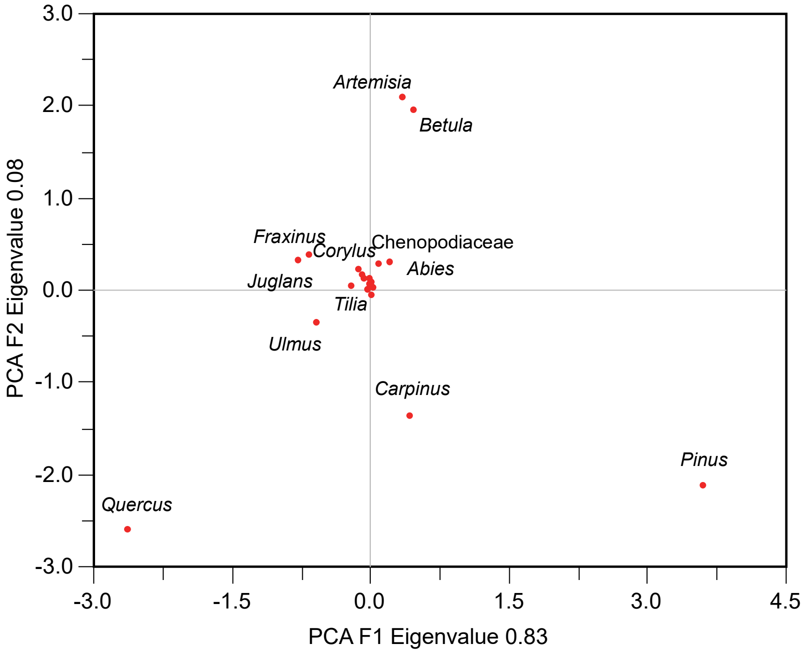

Supplementary Fig. 5. Results of principal components analysis (PCA) of the pollen percentage data from Lake Xiaolongwan. PCA was applied to the terrestrial pollen percentage data to extract the main gradient changes in the vegetation. All pollen taxa with a relative abundance >2% in at least two samples were used in the analysis. The first and second principal components (PCA F1 and PCA F2) have eigenvalues of 0.83 and 0.08, explaining 83% and 8% of the total variance of pollen data, respectively. Broadleaved taxa (*Quercus, Ulmus, Juglans* and *Fraxinus*) have negative loadings on axis 1, whereas temperate mixed deciduous and coniferous forest taxa (*Pinus, Abies, Betula*) and some herb taxa (e.g. *Artemisia* and Chenopodiaceae) have positive loadings. PCA F1 loadings represent a temperature-moisture gradient from warm-humid (negative) to cold-dry (positive) climate conditions.

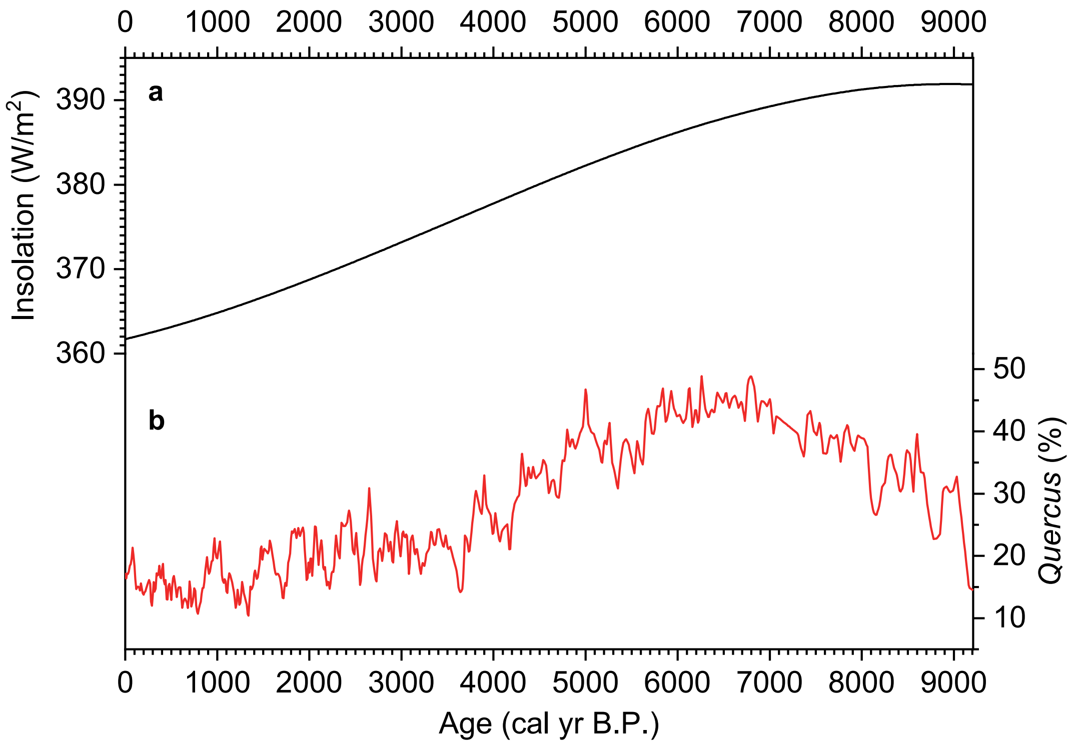

Supplementary Fig 6. A comparison of the record of *Quercus* percentages from Maar Lake Xiaolongwan and the 65ºN JJA insolation record. (**a**) JJA 65°N insolation. (**b**) *Quercus* percentages.

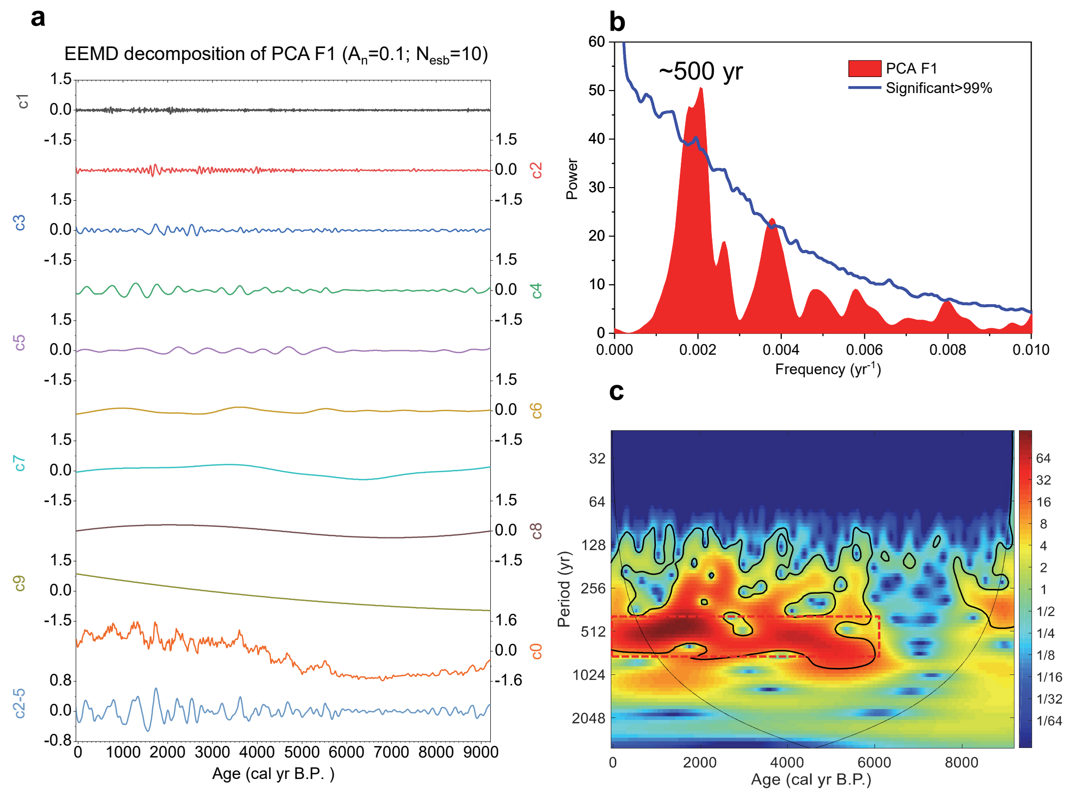

Supplementary Fig. 7. Results of time-series analysis of the PCA F1 sample scores. (**a**) EEMD [26, 27]: white noise (A_n) of 0.1 and the component number (N_{esb}) of 10≈($Log_2^{922}+1$) are used for the first EEMD component. Component c0 represents the original PCA F1 sample scores data. To remove high-frequency fluctuations and orbital and millennial trends, a new detrended time series was formed by summing components c2-c5 from the first decomposition (c0). (**b**) Results of univariate spectral analysis [28] of the PCA F1 sample scores time-series over the past 9260 yr. (**c**) Wavelet power spectrum [29] of PCA F1 sample scores. The 95% confidence level is outlined in black. The red dotted box indicates the strong oscillations of ~500-yr cycles after ~5700 cal yr B.P.

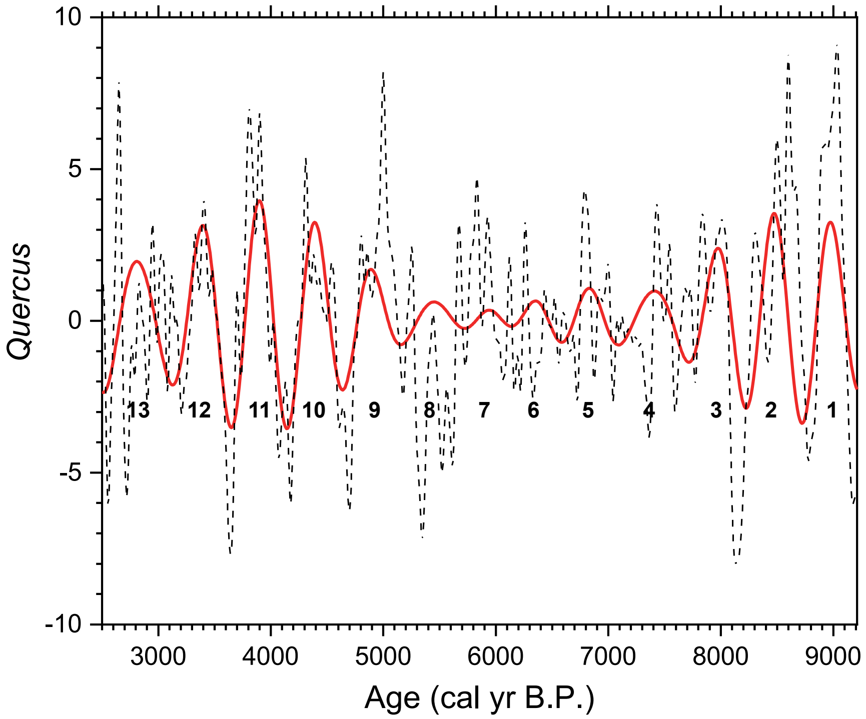

Supplementary Fig. 8. 400-600-yr band pass filter results for *Quercus* percentages. The black dotted curve shows the c2-c5 summed components of *Quercus* percentages. 400-600-yr band-pass filter results are shown by the red curve. The central frequencies and bandwidths of the *Quercus* percentages (c2-c5 component after HHT analysis) filters are 0.020 yr^{-1} (500-yr period), 0.017 yr^{-1} (600-yr period), and 0.025 yr^{-1} (400- yr period), respectively. The results show ~500-yr cycles.

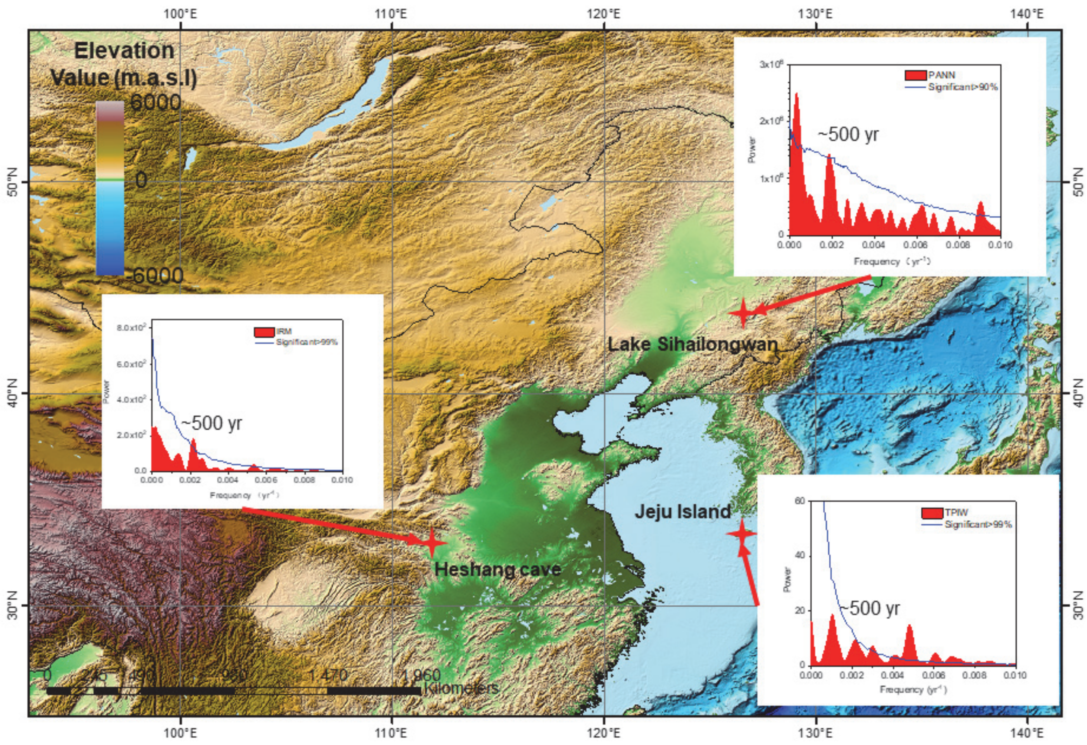

Supplementary Fig. 9. Locations of sites recording ~500-yr monsoonal climate cycles in East Asia and the corresponding results of univariate spectral analysis. The three red stars show the locations of Maar Lake Sihailongwan, Heshang cave and Jeju Island. Spectral analysis of pollen records from Maar Lake Sihailongwan [5] and Jeju Island in the Yellow Sea [6], and of the speleothems record from Heshang cave [7] in South China, show ~500-yr summer monsoon oscillations significant at the 90%, 99%, and 99% levels, respectively, during the mid-late Holocene. The map was generated using DIVA-GIS 7.5 (http://www.diva-gis.org/).

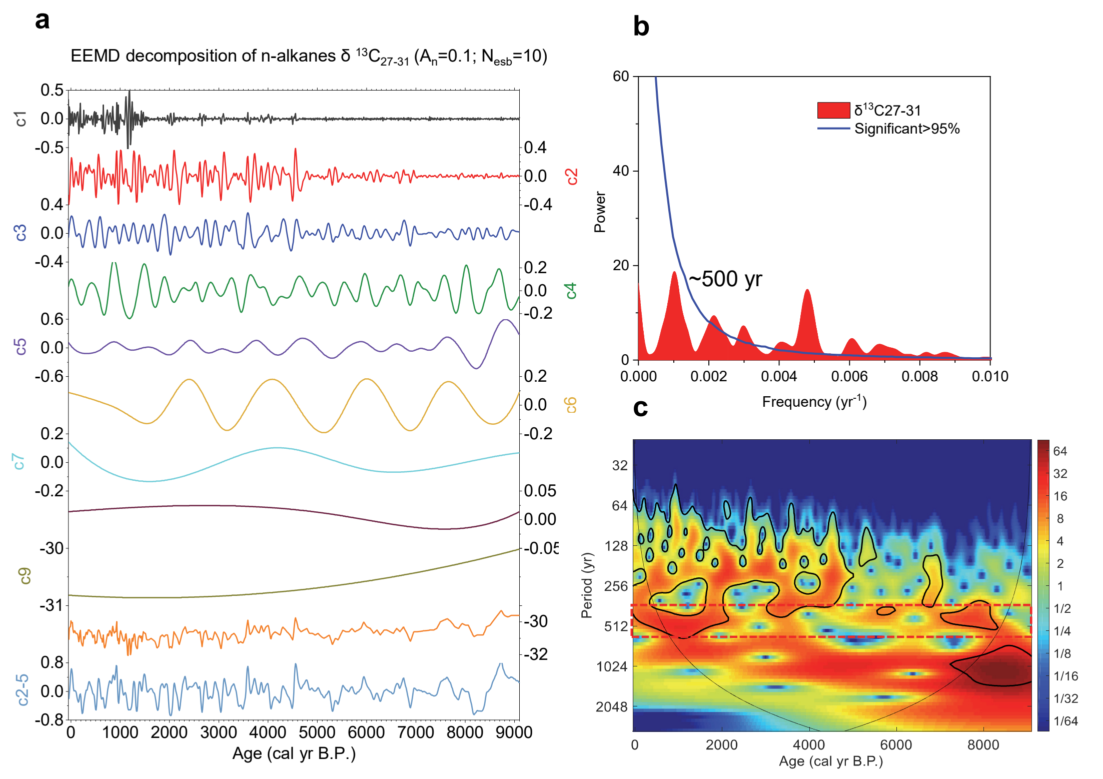

Supplementary Fig. 10. Results of time-series analysis of the record of long-chain n-alkanes ($\delta^{13}C_{27\text{-}31}$) for NE China. (**a**) EEMD[26, 27]: white noise (A_n) of 0.1 and the component number (N_{esb}) of 10≈ $(Log_2^{910} + 1)$ are used for the first EEMD component. Component c0 represents the original $\delta^{13}C_{27\text{-}31}$. To remove high-frequency fluctuations and orbital and millennial trends, a new detrended time series was produced by summing components c2-c5 from the first decomposition (c0). (**b**) Results of univariate spectral analysis[28] of the $\delta^{13}C_{27\text{-}31}$ time-series over the past 9100 yr. (**c**) Wavelet power spectrum[29] of $\delta^{13}C_{27\text{-}31}$. The 95% confidence level is outlined in black. The red dotted box highlights the ~500-yr periodicity.

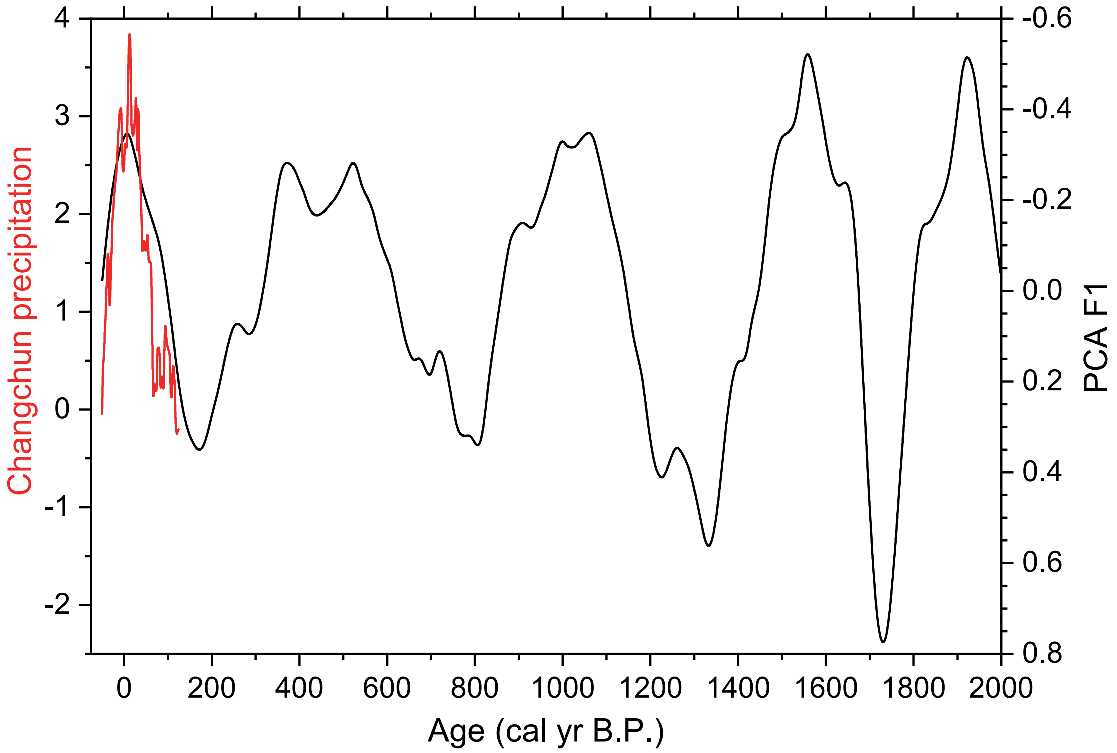

Supplementary Fig. 11. Comparison of PCA F1 sample scores and observed precipitation record for Changchun City. Black curve: detrended PCA F1 sample scores (c2-c5 summed components); red curve: residual mass curve of Changchun precipitation [30]. Precipitation changes in Changchun City are superimposed on the recent part of the PCA F1 record.

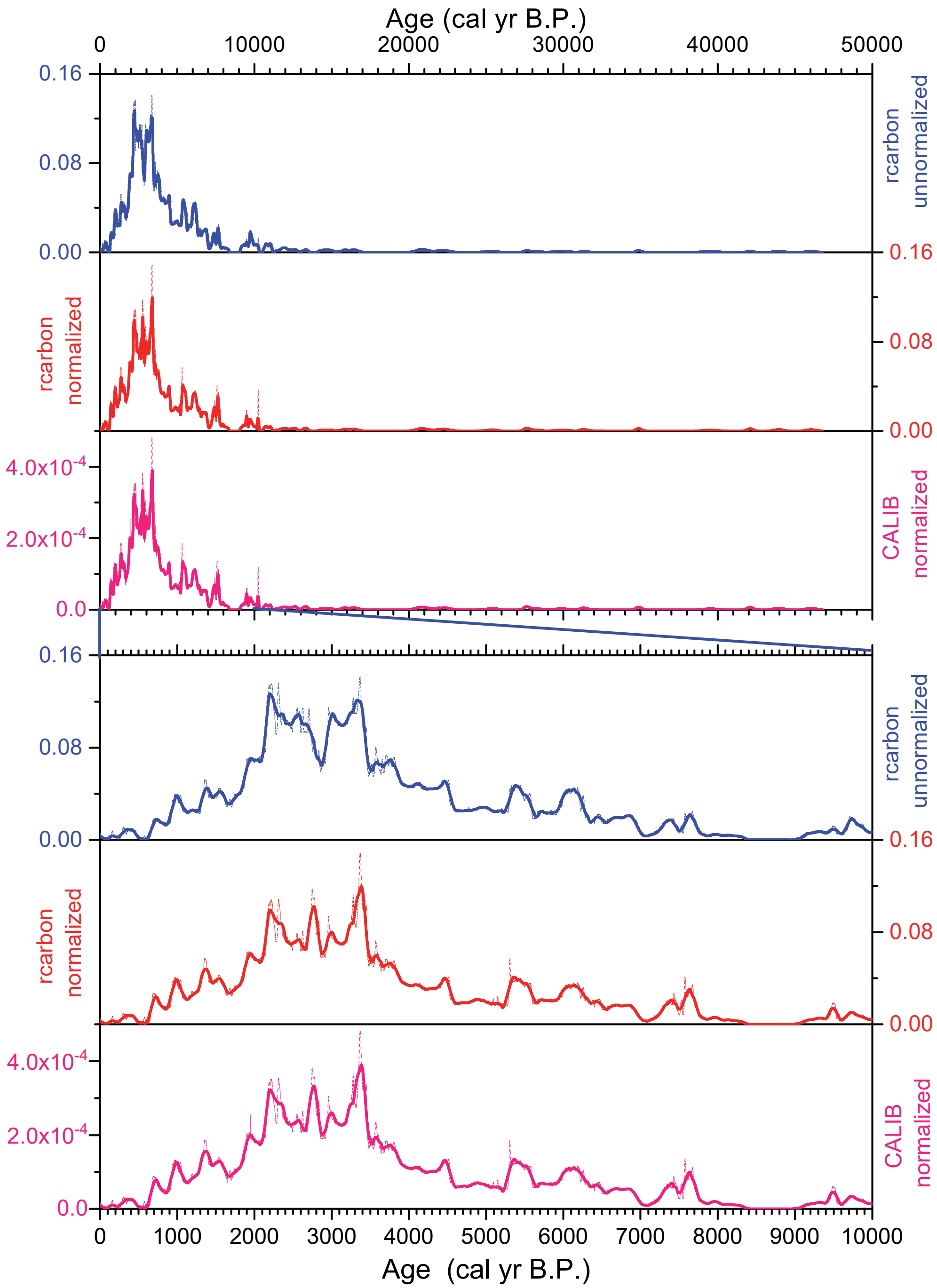

Supplementary Fig. 12. Comparison of SCP results between normalized and un-normalized radiocarbon dates using CALIB and R software with rcarbon packages. The blue, red and pink dotted curves show the SCP results using rcarbon unnormalized, rcarbon normalized and CALIB normalized methods, respectively. The results of 200-point-smoothing of the SCP using the three methods are shown by blue, red and pink curves, respectively.

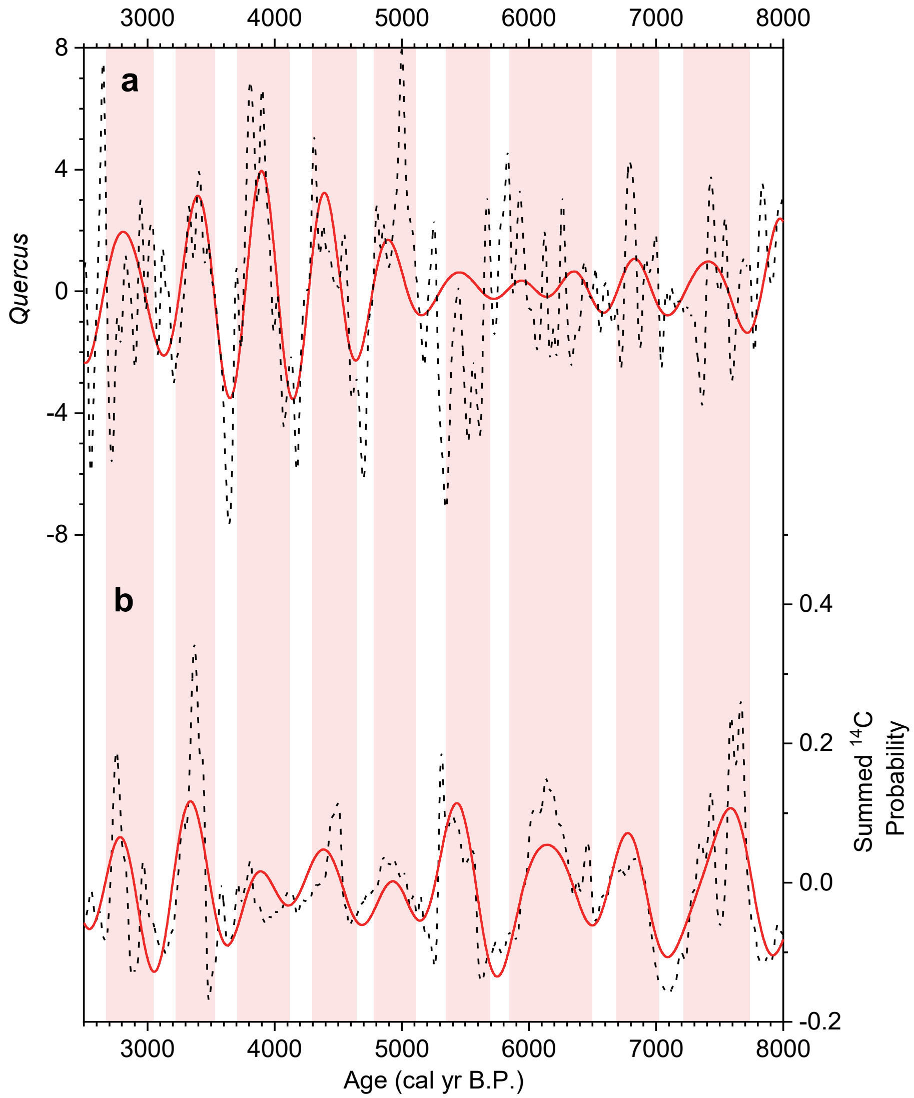

Supplementary Fig. 13. 400-600-year band pass filter results of *Quercus* percentages (EASM proxy) and 400-1250-year band pass filter results of SCP. (**a**) *Quercus* percentages. (**b**) Summed ^{14}C probability. The black dotted curves show the c2-c5 summed components of EASM and SCP, respectively. 400-600-yr band-pass filter results of the EASM and SCP are shown by the red curves. The central frequencies and bandwidths of the EASM filters are 0.020 yr^{-1} (500-yr period), 0.017 yr^{-1} (600-yr period), and 0.025 yr^{-1} (400-yr period), respectively. The central frequencies and bandwidths of the SCP (c2-c5 component after HHT analysis) filters are 0.020 yr^{-1} (500-yr period), 0.008 yr^{-1} (1250-yr period), and 0.025 yr^{-1} (400-yr period), respectively. Both proxies show ~500-yr cycles. The ~500-yr cyclical change in SCP is in-phase relative to ~500-yr oscillations in *Quercus* percentages during cycles 1-2 and 4-9, but lags by several decades during cycle 3 (around 6500 to 5500 cal yr B.P.).

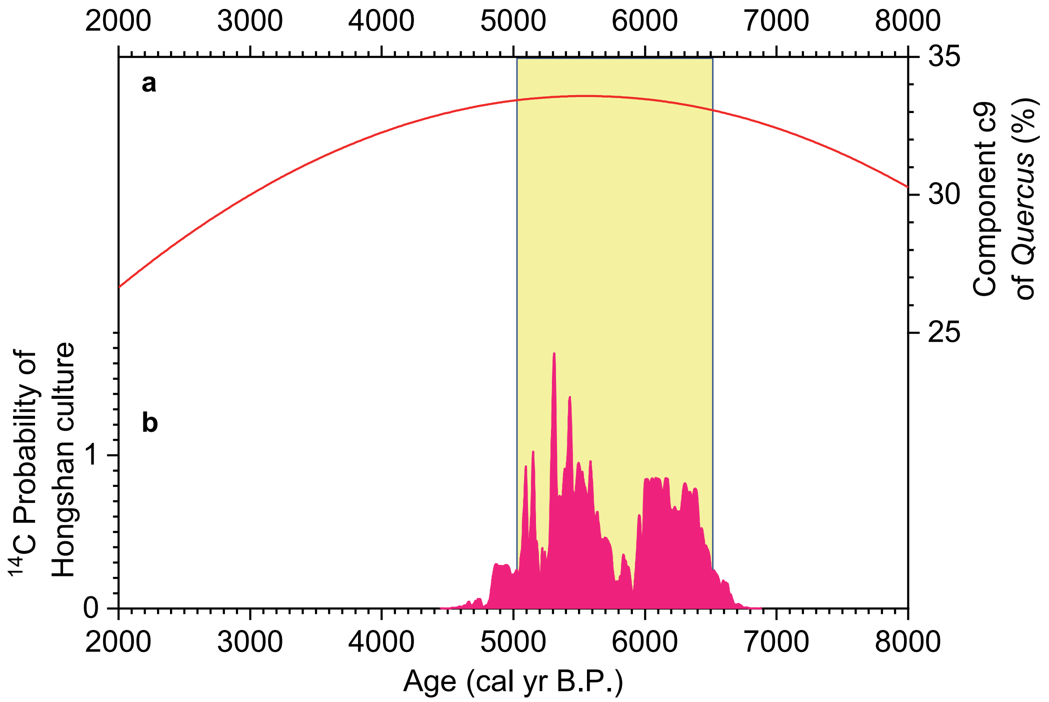

Supplementary Fig. 14. Comparison of the trend of *Quercus* percentages (component c9) with ^{14}C probability for the Hongshan culture. (**a**) Component c9 of *Quercus* percentages. (**b**) Density histogram of Hongshan culture ^{14}C probability. The yellow-shaded bar shows the optimum period of EASM development from ~6500 to 5000 cal yr B.P. which corresponds to a prosperous period of the Hongshan culture.

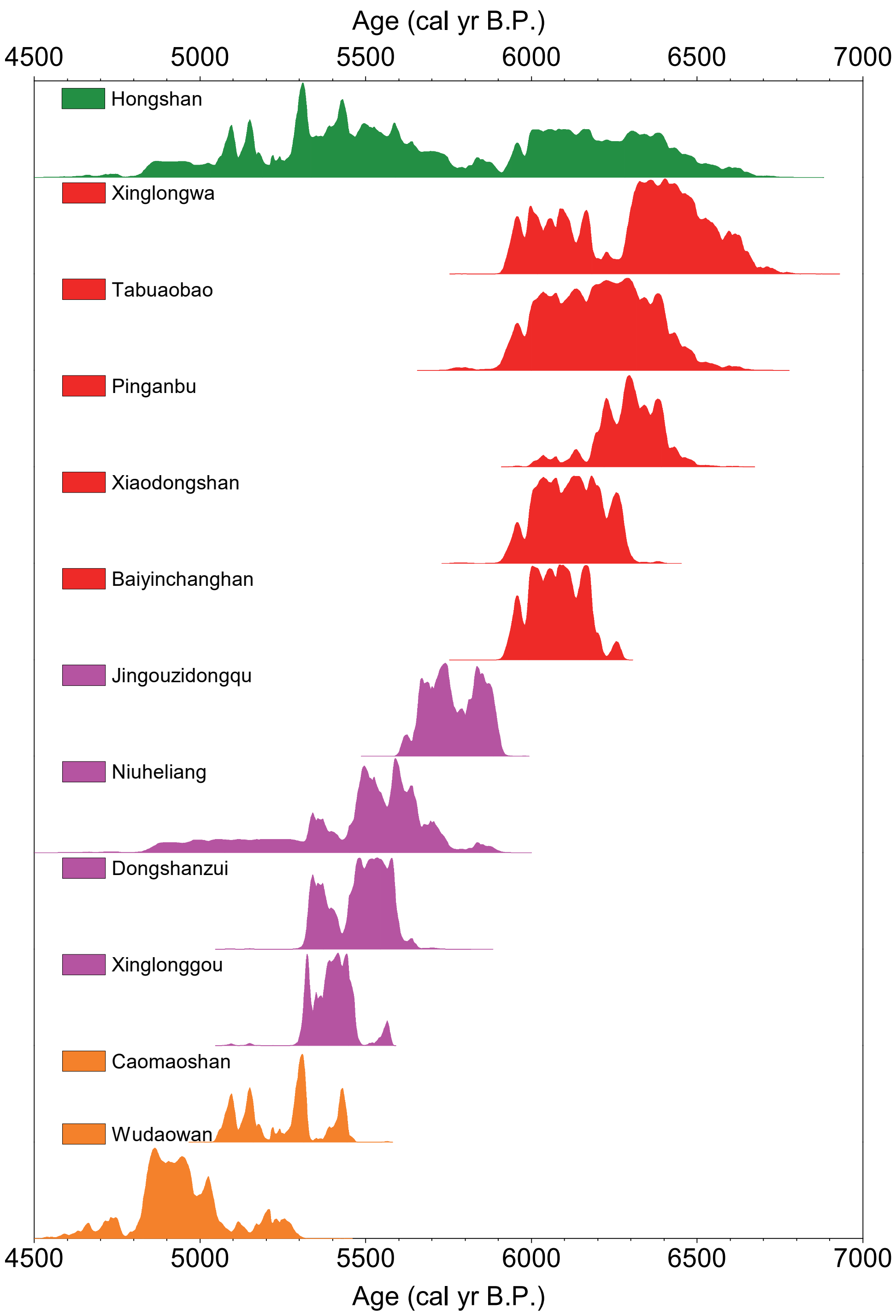

Supplementary Fig. 15. Density histogram of cultural ^{14}C probability from Hongshan prehistorical archeological sites.

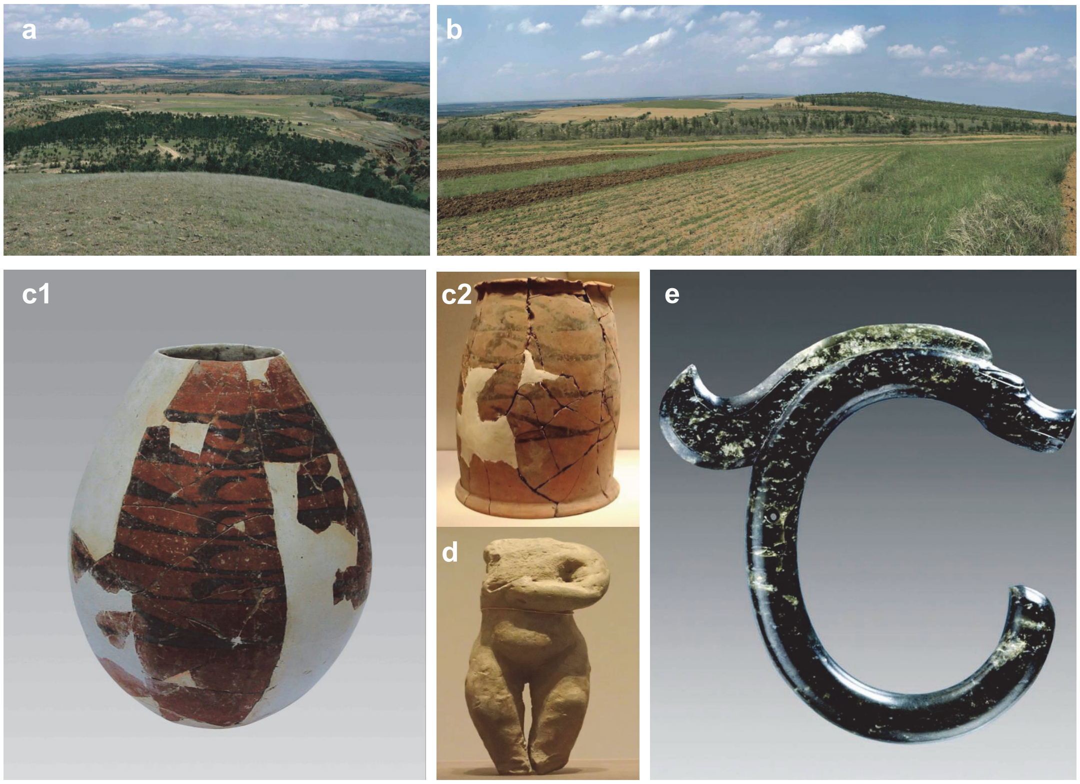

Supplementary Fig. 16. Examples of pottery and 'Jade pig loongs' (Yuzhulong) unearthed from sites of the Longshan Culture (**a**): Xinglongwa site. (**b**): Xinglonggou site. (**c1**) and (**c2**): Examples of pottery. (**d**): Pregnant female figure. (**e**): Jade pig loongs (Yuzhulong). The photos were taken by Yonggang Sun (**a**, **b**, **c1** and **e**) and Deke Xu (**c2** and **d**). Yonggang Sun authorized Houyuan Lu to use photos (**a**, **b**,**c1** and **e**) for this publication.

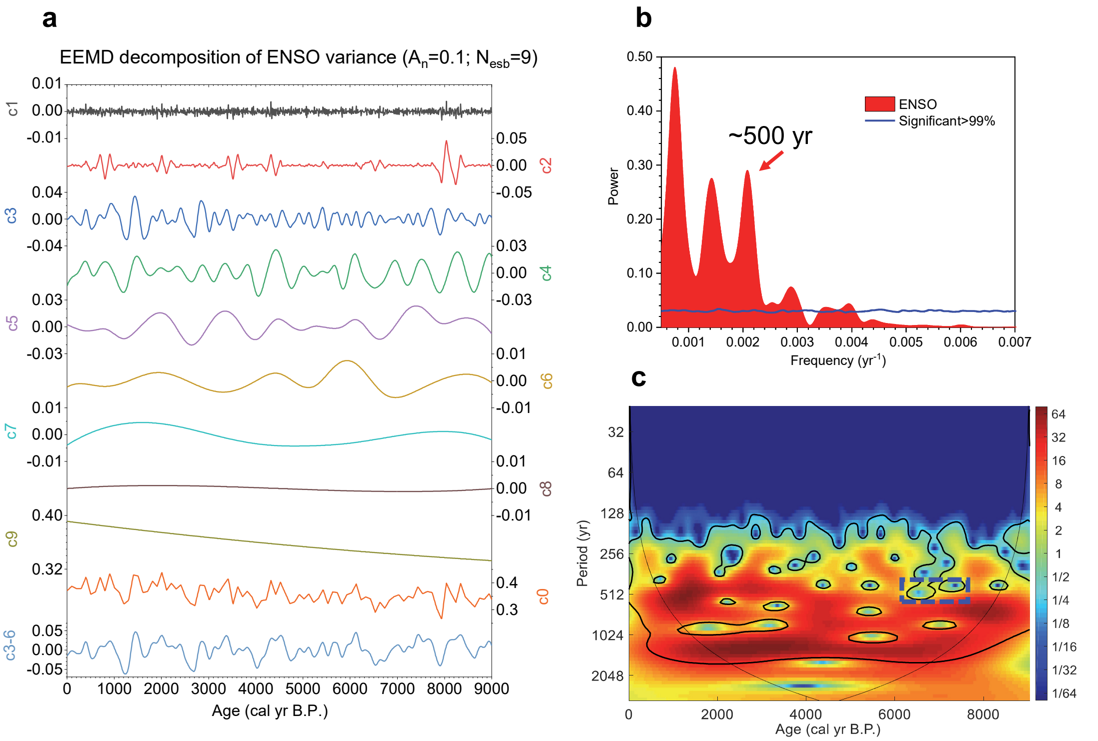

Supplementary Fig. 17. Results of time-series analysis of ENSO variance[7, 31]. (**a**) EEMD [26, 27]: white noise (A_n) of 0.1 and the component number (N_{esb}) of 10≈(Log_2^{905} + 1) are used for the first EEMD component. Component c0 represents the original ENSO variance data. In order to remove high-frequency fluctuations and orbital and millennial trends, a new detrended time series was generated by summing components c3-c6 from the first decomposition (c0). (**b**) Results of univariate spectral analysis [28] of the ENSO variance time-series over the past 9040 yr. (**c**) Wavelet power spectrum [29] of ENSO variance. The 95% confidence level is outlined in black. The blue dotted box indicates weak ~500-yr cycles during the period of 7500~5700 cal yr B.P.

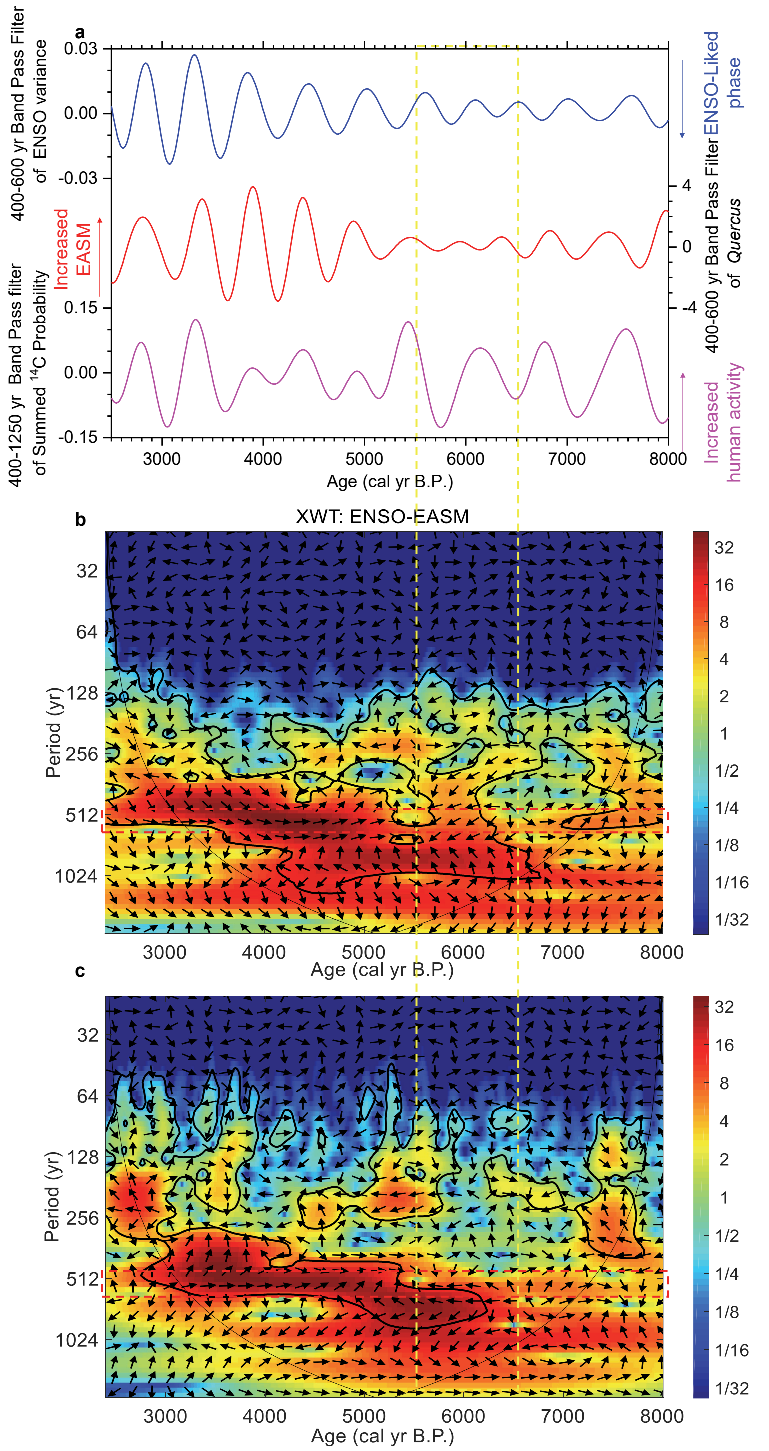

Supplementary Fig. 18. Filter, coherence and phase comparisons of ENSO, EASM and SCP. (a). Band pass filters of ENSO (c3-c6 component after HHT analysis) [7, 31] EASM (c2-c5 component after HHT analysis) and SCP (c2-c5 component after HHT analysis). The central frequencies and bandwidths of the SCP filters are 0.020 yr^{-1} (500-yr period), 0.008

yr^{-1} (1250-yr period), and 0.025 yr-1 (400-yr period), respectively. The central frequencies and bandwidths of the ENSO and EASM filters are 0.020 yr^{-1} (500-yr), 0.017 yr^{-1} (600-yr), and 0.0250 yr^{-1} (400-yr), respectively. The yellow-dashed box shows the period of low amplitude ~500-yr cycles between ENSO and EASM during 6500~5500 cal yr B.P.. **(b). Cross wavelet transform (XWT) power between ENSO and the EASM. (c). XWT power between the EASM and SCP.** The relative phase relationship is shown as arrows [nearly in-phase, pointing to the right (counter-clockwise from -90° up to 90°), lagging or out of phase, and pointing to the left (counter-clockwise from 90° down to -90°)]. During 6500~5500 cal yr B.P. (the yellow-shaded bar) there was a lagged or out of phase relationship of the ~500-yr periodicity between ENSO and the EASM, and between EASM and SCP. The two red-dashed rectangles highlight the ~500-yr periodicity.

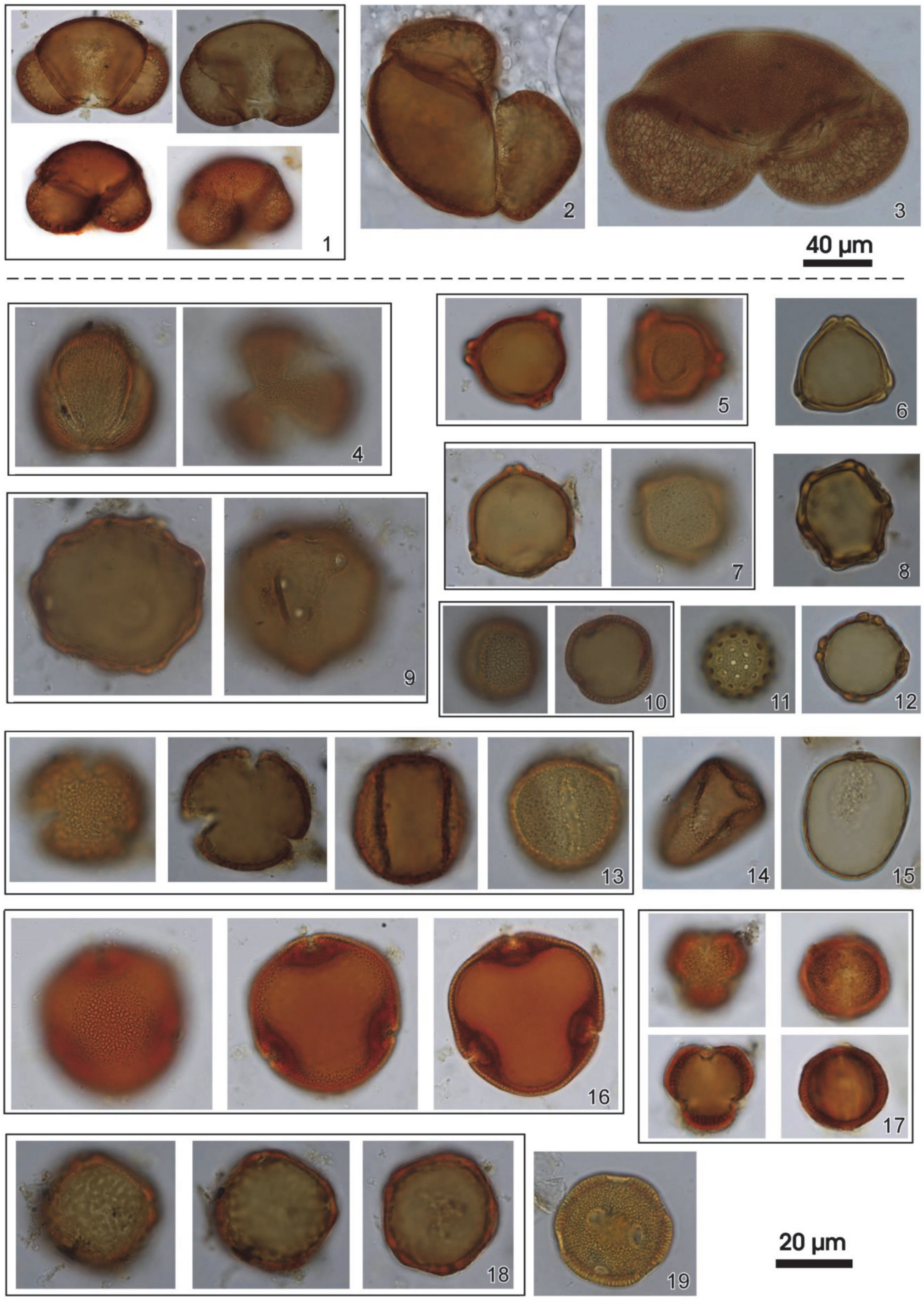

Supplementary Fig. 19. Common pollen types in Lake Xiaolongwan.

Captions: 1. *Pinus*, 2. *Abies*, 3. *Picea*, 4. *Acer*, 5. *Betula*, 6. *Corylus*, 7. *Carpinus*, 8. *Alnus*, 9. *Juglans*, 10. *Fraxinus*, 11. Chenopodiaceae, 12. *Myriophyllum*, 13. *Quercus*, 14. Cyperaceae, 15. Poaceae, 16. *Tilia*, 17. *Artemisia*, 18. *Ulmus*, 19. Caryophyllaceae.

Supplementary Table

Supplementary Table 1. AMS ^{14}C ages for the sediment core from Lake Xiaolongwan

The reservoir correction factor is 212 yr for the uppermost 20 cm of the core. All corrected ^{14}C ages with 2σ-range were calibrated using the IntCal13 data set using the CALIB 7.1 program [22, 23].

Lab No.	Depth	Dated material	Radiocarbon Age	Corrected ^{14}C age	Uncertainty	Calibrated age	2σ-range
	(cm)		(yr B.P.)	(yr B.P.)	yr	(Cal yr B.P.)	yr
BA07706	20	Bulk	400	188	±40	179	1-305
BA07707	56	Leaf	1215	1003	±40	922	796-978
BA06527	101	Wood	1875	1663	±40	1569	1417-1695
BA07708	154	Leaf	2615	2403	±40	2440	2345-2698
BA07709	165	Leaf	2880	2668	±40	2780	2744-2850
CG-4761	186	Bulk	3490	3278	±90	3513	3265-3811
BA07710	219	Leaf	3850	3638	±40	3954	3850-4084
CG-4762	266	Bulk	4780	4568	±80	5222	4972-5570
BA07711	280	Leaf	4840	4628	±35	5407	5299-5465
Poz-42550	310	Leaf	6230	6018	±35	6860	6753-6950

Supplementary References

1. Chu G*, et al.* Dinocyst microlaminations and freshwater "red tides" recorded in Lake Xiaolongwan, northeastern China. *J Paleolimnol* **39**, 319-333 (2008).
2. Sun Q*, et al.* Alkanes, compound-specific carbon isotope measures and climate variation during the last millennium from varved sediments of Lake Xiaolongwan, northeast China. *J Paleolimnol* **50**, 331-344 (2013).
3. Chu GQ*, et al.* A 1600 year multiproxy record of paleoclimatic change from varved sediments in Lake Xiaolongwan, northeastern China. *J Geophys Res-Atmos* **114**, D22108 (2009).
4. Xu D*, et al.* 500-year climate cycles stacking of recent centennial warming documented in an East Asian pollen record. *Sci Rep* **4**, 3611 (2014).

5. Stebich M, Rehfeld K, Schlütz F, Tarasov PE, Liu J, Mingram J. Holocene vegetation and climate dynamics of NE China based on the pollen record from Sihailongwan Maar Lake. *Quaternary Sci Rev* **124**, 275-289 (2015).
6. Park J. Solar and tropical ocean forcing of late-Holocene climate change in coastal East Asia Regional. *Palaeogeogr Palaeocl* **469**, 74-83 (2017).
7. Zhu Z*, et al.* Holocene ENSO-related cyclic storms recorded by magnetic minerals in speleothems of central China. *Proc Natl Acad Sci U S A* **114**, 852-857 (2017).
8. Chu G*, et al.* Holocene cyclic climatic variations and the role of the Pacific Ocean as recorded in varved sediments from northeastern China. *Quaternary Sci Rev* **102**, 85-95 (2014).
9. Surovell TA, Finley JB, Smith GM, Brantingham PJ, Kelly R. Correcting temporal frequency distributions for taphonomic bias. *J Archaeol Sci* **36**, 1715-1724 (2009).
10. Gamble C, Davies W, Pettitt P, Hazelwood L, Richards M. The Archaeological and Genetic Foundations of the European Population during the Late Glacial: Implications for 'Agricultural Thinking'. *Cambridge Archaeological Journal* **15**, 193-223 (2005).
11. Shennan S, Edinborough K. Prehistoric population history: from the Late Glacial to the Late Neolithic in Central and Northern Europe. *J Archaeol Sci* **34**, 1339-1345 (2007).
12. Williams AN. The use of summed radiocarbon probability distributions in archaeology: a review of methods. *J Archaeol Sci* **39**, 578-589 (2012).
13. Chaput MA, Gajewski K. Radiocarbon dates as estimates of ancient human population size. *Anthropocene* **15**, 3-12 (2016).
14. Brown WA. Through a filter, darkly: population size estimation, systematic error, and random error in radiocarbon-supported demographic temporal frequency analysis. *J Archaeol Sci* **53**, 133-147 (2015).
15. Surovell TA, Brantingham PJ. A note on the use of temporal frequency distributions in studies of prehistoric demography. *J Archaeol Sci* **34**, 1868-1877 (2007).
16. Rick JW. Dates as Data: An Examination of the Peruvian Preceramic Radiocarbon Record. *American Antiquity* **52**, 55-73 (1987).
17. Wang C, Lu H, Zhang J, Gu Z, He K. Prehistoric demographic fluctuations in China inferred from radiocarbon data and their linkage with climate change over the past 50,000 years. *Quaternary Sci Rev* **98**, 45-59 (2014).

18. Maher LA, Banning EB, Chazan M. Oasis or Mirage? Assessing the Role of Abrupt Climate Change in the Prehistory of the Southern Levant. *Cambridge Archaeological Journal* **21**, 1-30 (2011).

19. Ward GK, Wilson SR. Ward G K, Wilson S R. Procedures for comparing and combining radiocarbon age determinations: a critique. *Archaeometry* **20**, 19-31 (1978).

20. Selden RZ. Modeling Regional Radiocarbon Trends: A Case Study from the East Texas Woodland Period. *Radiocarbon* **54**, 239-265 (2012).

21. Ramsey CB. Bayesian Analysis of Radiocarbon Dates. *Radiocarbon* **51**, 337-360 (2009).

22. Reimer PJ*, et al.* Intcal13 and Marine13 Radiocarbon Age Calibration Curves 0-50,000 Years Cal Bp. *Radiocarbon* **55**, 1869-1887 (2013).

23. Stuiver M*, et al.* INTCAL98 radiocarbon age calibration, 24,000-0 cal BP. *Radiocarbon* **40**, 1041-1083 (1998).

24. Weninger B, Clare L, Jöris O, Jung R, Edinborough K. Quantum theory of radiocarbon calibration. *World Archaeology* **47**, 543-566 (2015).

25. Bamforth DB, Grund B. Radiocarbon calibration curves, summed probability distributions, and early Paleoindian population trends in North America. *J Archaeol Sci* **39**, 1768-1774 (2012).

26. Wu Z, Huang NE. Ensemble Empirical Mode Decomposition: A Noise-Assisted Data Analysis Method. *Advances in Adaptive Data Analysis* **01**, 1-41 (2009).

27. Huang NE*, et al.* The empirical mode decomposition and the Hilbert spectrum for nonlinear and non-stationary time series analysis. *P Roy Soc A-Math Phy* **454**, 903-995 (1998).

28. Schulz M, Mudelsee M. REDFIT: estimating red-noise spectra directly from unevenly spaced paleoclimatic time series. *Comput Geosci-Uk* **28**, 421-426 (2002).

29. Torrence C, Compo GP. A practical guide to wavelet analysis. *B Am Meteorol Soc* **79**, 61-78 (1998).

30. Li W, Guo Y. Analysis on precipitation change in Changchun city. *Jilin Water Resources* **308**, 33-34 (2008).

31. Liu Z, Lu Z, Wen X, Otto-Bliesner BL, Timmermann A, Cobb KM. Evolution and forcing mechanisms of El Niño over the past 21,000 years. *Nature* **515**, 550 (2014).
