# Supplementary material for: Synchronous 500-year oscillations of monsoon climate and human activity in Northeast Asia
Source: Nat Commun. 2019 Sep 11;10:4105. doi: 10.1038/s41467-019-12138-0 (PMC6739325; doi:10.1038/s41467-019-12138-0)
Supplement: Supplementary file 2 — Reporting Summary [file 41467_2019_12138_MOESM2_ESM.pdf]

nature research

Corresponding author(s): Deke Xu and Houyuan Lu

Last updated by author(s): Aug 17, 2019

Reporting Summary

Nature Research wishes to improve the reproducibility of the work that we publish. This form provides structure for consistency and transparency in reporting. For further information on Nature Research policies, see Authors & Referees and the Editorial Policy Checklist .

Statistics

For all statistical analyses, confirm that the following items are present in the figure legend, table legend, main text, or Methods section.

n/a	Confirmed	
☒	☐	The exact sample size (*n*) for each experimental group/condition, given as a discrete number and unit of measurement
☒	☐	A statement on whether measurements were taken from distinct samples or whether the same sample was measured repeatedly
☒	☐	The statistical test(s) used AND whether they are one- or two-sided<br>*Only common tests should be described solely by name; describe more complex techniques in the Methods section.*
☒	☐	A description of all covariates tested
☒	☐	A description of any assumptions or corrections, such as tests of normality and adjustment for multiple comparisons
☒	☐	A full description of the statistical parameters including central tendency (e.g. means) or other basic estimates (e.g. regression coefficient) AND variation (e.g. standard deviation) or associated estimates of uncertainty (e.g. confidence intervals)
☒	☐	For null hypothesis testing, the test statistic (e.g. *F*, *t*, *r*) with confidence intervals, effect sizes, degrees of freedom and *P* value noted<br>*Give P values as exact values whenever suitable.*
☒	☐	For Bayesian analysis, information on the choice of priors and Markov chain Monte Carlo settings
☒	☐	For hierarchical and complex designs, identification of the appropriate level for tests and full reporting of outcomes
☒	☐	Estimates of effect sizes (e.g. Cohen's *d*, Pearson's *r*), indicating how they were calculated

Our web collection on statistics for biologists contains articles on many of the points above.

Software and code

Policy information about availability of computer code

Data collection	No software was used.
Data analysis	Matlab 2018a, R 3.5.2, Tilia 2.1.1 and Origin 2018 were used.

For manuscripts utilizing custom algorithms or software that are central to the research but not yet described in published literature, software must be made available to editors/reviewers. We strongly encourage code deposition in a community repository (e.g. GitHub). See the Nature Research guidelines for submitting code & software for further information.

Data

Policy information about availability of data

All manuscripts must include a data availability statement. This statement should provide the following information, where applicable:

- Accession codes, unique identifiers, or web links for publicly available datasets
- A list of figures that have associated raw data
- A description of any restrictions on data availability

The source data underlying Fig. 2-5, and Supplementary Figs. 7-8 and 10-15 are provided as Supplementary Dataset files.

Field-specific reporting

Please select the one below that is the best fit for your research. If you are not sure, read the appropriate sections before making your selection.

☐ Life sciences ☐ Behavioural & social sciences ☒ Ecological, evolutionary & environmental sciences

For a reference copy of the document with all sections, see nature.com/documents/nr-reporting-summary-flat.pdf

Ecological, evolutionary & environmental sciences study design

All studies must disclose on these points even when the disclosure is negative.

Study description	We used pollen data to reconstruct the paleovegetation and paleoclimate change. We used radiocarbon dates to reconstruct human activity. Then we tried to find their relationship.
Research sample	We collected and analyzed the pollen data and radiocarbon dates.
Sampling strategy	We counted an average of 701 (range 585-949) terrestrial pollen grains for each sample. The statistic has been much larger than the requirement for 300 grains in the temperate region. We assembled 627 archaeological radiocarbon dates to build a dataset for NE China (~1.52 million km2) . The number of dates collected from NE China exceed the minimum size of 500 samples for a large region (~7.69 million km2) (65 samples in an area of 1 million km2), and therefore large sampling can be treated as random sampling and mitigate site- and period- level biases.
Data collection	Deke Xu identified and analyzed the pollen data. Can Wan, Houyuan Lu and Deke Xu compiled archeological radiocarbon dates.
Timing and spatial scale	The collection of pollen data and radiocarbon dates started at September 2010 and ended in June 2018.
Data exclusions	Radiocarbon dates with the following attributes were excluded: (1) Dates with large error bars (1σ standard deviation >400 14C year); (2) dates from shells, soils, unknown materials or other materials considered inappropriate for dating; and (3) those dates derived from sites or materials that had weak associations with human occupation or settlement, such as ancient temples, pagodas or canoes.
Reproducibility	All attempts to repeat the experiment were successful.
Randomization	We assembled 627 archeological radiocarbon dates to build a dataset for NE China. These dates from NE China (~1.52 million km2) exceed the minimum size of 500 samples for a large region (~7.69 million km2) (65 samples in an area of 1 million km2), and therefore large sampling can be treated as random sampling and mitigate site- and period- level biases.
Blinding	Pollen analysis can't be applied to blinding experiments.

Did the study involve field work? ☒ Yes ☐ No

Field work, collection and transport

Field conditions	In the early spring of 2006, we obtained cores from the frozen lake. The air temperature is below 0℃ and the lake temperature below the ice is around 4℃.
Location	Maar Lake Xiaolongwan is located in the Changbai Mountains, Jilin Province, NE China. (42°18.0′N, 126°21.5′E, altitude: 655 masl, water depth of 15 m)
Access and import/export	The Longwan National Park authorized us to sample in Lake Xiaolongwan.
Disturbance	The corer was carefully operated and allowed to slowly penetrate the sediment by gravity. After cores were recovered, water in the upper part of the cores was removed by syringe and dried by inserting paper towels. Cores were kept vertically during transportation from the field to lab. In the lab, the cores were dried further by inserting paper towels before the cores were cut. All the samples in these cores have not been disturbed using this method.

Reporting for specific materials, systems and methods

We require information from authors about some types of materials, experimental systems and methods used in many studies. Here, indicate whether each material, system or method listed is relevant to your study. If you are not sure if a list item applies to your research, read the appropriate section before selecting a response.

Materials & experimental systems

n/a	Involved in the study	
☒	☐	Antibodies
☒	☐	Eukaryotic cell lines
☒	☐	Palaeontology
☒	☐	Animals and other organisms
☒	☐	Human research participants
☒	☐	Clinical data

Methods

n/a	Involved in the study	
☒	☐	ChIP-seq
☒	☐	Flow cytometry
☒	☐	MRI-based neuroimaging
