# Supplementary material for: Synchronous 500-year oscillations of monsoon climate and human activity in Northeast Asia
Source: Nat Commun. 2019 Sep 11;10:4105. doi: 10.1038/s41467-019-12138-0 (PMC6739325; doi:10.1038/s41467-019-12138-0)
Supplement: Supplementary file 3 — Description of Additional Supplementary Files [file 41467_2019_12138_MOESM3_ESM.pdf]

Description of Supplementary Data

File Name: Supplementary Data 1

Description: The time series and statistical analysis results of pollen data

This file include:

Pollen percentages (sheet1): Pollen percentages of lake Xiaolongwan

***Quercus* percentage eemd** (sheet2): Hilbert-Huang Transform Ensemble empirical mode decompositions (eemd) of *Quercus* percentages

PCA F1 eemd (sheet3): Hilbert-Huang Transform eemd of PCA F1

Bandpass filter (sheet4): 400-600 year bandpass filter of *Quercus* percentages

File Name: Supplementary Data 2

Description: The dataset of integrated archaeological radiocarbon dates from NE China

This file include:

^{14}C dates in NE China (sheet1): 627 archaeological radiocarbon dates in NE China

^{14}C ages and occupations (sheet2): ^{14}C ages and ^{14}C occupations from archaeological sites in NE China

Summed ^{14}C probability eemd (sheet3): Hilbert-Huang Transform eemd of summed ^{14}C probability

Bandpass filter (sheet4): 400-1250 year bandpass filter of summed ^{14}C probability

Culture ^{14}C NE China (sheet5): Integrated ^{14}C dates of Hongshan Culture
